# Effect of a scaled-up neonatal resuscitation quality improvement package on intrapartum-related mortality in Nepal: A stepped-wedge cluster randomized controlled trial

**DOI:** 10.1371/journal.pmed.1002900

**Published:** 2019-09-09

**Authors:** Ashish KC, Uwe Ewald, Omkar Basnet, Abhishek Gurung, Sushil Nath Pyakuryal, Bijay Kumar Jha, Anna Bergström, Leif Eriksson, Prajwal Paudel, Sushil Karki, Sunil Gajurel, Olivia Brunell, Johan Wrammert, Helena Litorp, Mats Målqvist

**Affiliations:** 1 Department of Women’s and Children’s Health; Uppsala University, Uppsala, Sweden; 2 Society of Public Health Physician Nepal, Kathmandu, Nepal; 3 Golden Community, Jawgal, Lalitpur, Nepal; 4 Nepal Health Research Council, RamshahPath, Kathmandu, Nepal; 5 Ministry of Health and Population, Government of Nepal, Kathmandu, Nepal; 6 UCL Institute for Global Health (IGH), University College London, London, United Kingdom; 7 Department of Public Health and Caring Sciences, Uppsala University, Uppsala, Sweden; 8 Life Line Nepal, Kathmandu, Nepal; 9 Kamana Health Nepal, Kathmandu, Nepal; Stellenbosch University, SOUTH AFRICA

## Abstract

**Background:**

Improving quality of intrapartum care will reduce intrapartum stillbirth and neonatal mortality, especially in resource-poor settings. Basic neonatal resuscitation can reduce intrapartum stillbirth and early neonatal mortality, if delivered in a high-quality health system, but there is a dearth of evidence on how to scale up such evidence-based interventions. We evaluated the scaling up of a quality improvement (QI) package for neonatal resuscitation on intrapartum-related mortality (intrapartum stillbirth and first day mortality) at hospitals in Nepal.

**Methods and findings:**

We conducted a stepped-wedge cluster randomized controlled trial in 12 hospitals over a period of 18 months from April 14, 2017, to October 17, 2018. The hospitals were assigned to one of four wedges through random allocation. The QI package was implemented in a stepped-wedge manner with a delay of three months for each step. The QI package included improving hospital leadership on intrapartum care, building health workers’ competency on neonatal resuscitation, and continuous facilitated QI processes in clinical units. An independent data collection system was set up at each hospital to gather data on mortality through patient case note review and demographic characteristics of women using semi-structured exit interviews. The generalized linear mixed model (GLMM) and multivariate logistic regression were used for analyses. During this study period, a total of 89,014 women–infant pairs were enrolled. The mean age of the mother in the study period was 24.0 ± 4.3 years, with 54.9% from disadvantaged ethnic groups and 4.0% of them illiterate. Of the total birth cohort, 54.4% were boys, 16.7% had gestational age less than 37 weeks, and 17.1% had birth weight less than 2,500 grams. The incidence of intrapartum-related mortality was 11.0 per 1,000 births during the control period and 8.0 per 1,000 births during the intervention period (adjusted odds ratio [aOR], 0.79; 95% CI, 0.69–0.92; *p* = 0.002; intra-cluster correlation coefficient [ICC], 0.0286). The incidence of early neonatal mortality was 12.7 per 1,000 live births during the control period and 10.1 per 1,000 live births during the intervention period (aOR, 0.89; 95% CI, 0.78–1.02; *p* = 0.09; ICC, 0.1538). The use of bag-and-mask ventilation for babies with low Apgar score (<7 at 1 minute) increased from 3.2% in the control period to 4.0% in the intervention period (aOR, 1.52; 95% CI, 1.32–1.77, *p* = 0.003). There were two major limitations to the study; although a large sample of women–infant pairs were enrolled in the study, the clustering reduced the power of the study. Secondly, the study was not sufficiently powered to detect reduction in early neonatal mortality with the number of clusters provided.

**Conclusion:**

These results suggest scaled-up implementation of a QI package for neonatal resuscitation can reduce intrapartum-related mortality and improve clinical care. The QI intervention package is likely to be effective in similar settings. More implementation research is required to assess the sustainability of QI interventions and quality of care.

**Trial registration:**

ISRCTN30829654.

## Abstract 2

Ashish KC and colleagues report on their trial for improving neonatal survival and clinical care with an improved resuscitation protocol in Nepal.

## Author summary

**Why was this study done?:**

- Most studies on the quality improvement (QI) interventions for neonatal resuscitation have focused on a set of microlevel intervention to improve health workers’ performance.
- There have been fewer QI packages for neonatal resuscitation with a mix of meso- (improving hospital leadership and governance) and microlevel interventions.
- Very little is known on the effect of a QI package for implementing neonatal resuscitation on the quality of intrapartum care and survival.

**What did the researchers do and find?:**

- We sought to evaluate the effectiveness of a package of QI interventions targeting neonatal resuscitation on quality of care and survival in 12 public hospitals of Nepal in an 18-month period in a stepped-wedge trial.
- During the study period, 40,258 women delivered during the control period and 49,004 delivered during the intervention period. After adjusting for clustering, there was a significant reduction of intrapartum related deaths (intrapartum stillbirths and first day mortality) in the intervention period (aOR, 0.79; 95% CI, 0.69–0.92).
- The use of bag-and-mask ventilation for all newborns as well as for newborns with an Apgar score of <7 at 1 minute increased from 2.5% to 5.4%, respectively, in the intervention period as compared with before the intervention.

**What do these findings mean?:**

- These findings suggest that a set of meso- and microlevel QI interventions implemented adequately can improve the quality of neonatal resuscitation care and reduce intrapartum-related mortality.
- The findings call for more attention to implement a QI package that aims to improve the leadership accountability towards care such that a conducive environment is created for health workers to implement the technical skills.
- Although QI intervention is likely to be effective, more implementation research is required to assess the sustainability of QI interventions and the quality of intrapartum care.

## Introduction

Achieving Sustainable Development Goal (SDG) 3.2, to reduce global neonatal mortality to 12 per 1,000 live births by 2030, will require transformative changes in healthcare systems, with increased efforts to deliver high-quality services [1,2,3,4]. In the last 20 years, significant investment has been made to improve the behavior of communities when it comes to seeking pregnancy and delivery care at health institutions [5, 6]. As a result, globally, in 2018, almost 59% of women received all four antenatal checkups, and 75% of women were delivered by a skilled provider [7]. However, health institutions have not been able to step up the pace in delivering high-quality and trustworthy care to women and families [8]. In the SDG era, a healthcare system consistently delivering optimal and consistent healthcare remains to be a centerpiece, not only to accelerate the momentum for improving health outcomes but also to garner trust through positive user experience [9, 10]. In 2017, poor quality of care accounted for almost 1 million neonatal deaths, mostly during the intrapartum period [11]. Improving care during labor and birth will have the highest impact on survival, as it is the period of highest risk to mothers and newborns, with almost 2.2 million intrapartum-related mortality occurring during this period every year [12,13].

Improving the quality of intrapartum care requires investment not only in human resources, equipment, and infrastructure to prepare facilities for care provision. Multilayered quality improvement (QI) efforts at the macro (national) [14], meso (subnational or hospital), and micro (health worker) levels are also needed [15,16]. To achieve a multilayered QI intervention, there is a need for strengthened governance and leadership structures facilitating a process of accountability towards better care at all levels.

Similar to the other low-income countries in South Asia, Nepal has witnessed an unprecedented increase in the utilization of health institutions for maternal and newborn care [17]. As a result of this, two thirds of the deliveries are now taking place in health institutions; nevertheless, stillbirth and neonatal death still occur due to poor-quality care [18,19]. Improving the quality of care at health institutions through improved governance for care has been an evidence-based solution [20,21,22].

A systematic review of the effect of QI packages for neonatal resuscitation has shown more than 2-fold improvement in early initiation of bag-and-mask ventilation in nonbreathing babies [23]. We have previously conducted a study to evaluate a QI package for neonatal resuscitation at a tertiary hospital in Kathmandu [24]. This QI package displayed a large effect on intrapartum-related mortality over the nine-month follow-up (adjusted odds ratio [aOR], 0.46; CI 95% 0.32–0.66) [25]. Based on reviews of QI interventions and our previous learning from this study [26], we hypothesized that bringing change to any healthcare setting requires four levers for change [25,27]: firstly, improving leadership accountability for high-quality care for mothers and newborns [28]; secondly, setting up mechanisms for continuous QI processes with a Plan-Do-Study-Act (PDSA) approach [29], facilitation[30], and audit feedback [31] in the healthcare institution and labor units; thirdly, introducing new standards and techniques for improving care[32]; and finally, setting up a quality metric system for change in the process and outcome of care [33].

Based on these findings, the Ministry of Health and Population, Nepal, developed a QI package, Nepal Perinatal Quality Improvement Package (NePeriQIP) to be implemented in 12 public hospitals in Nepal (S1 Text) [34]. The aim of the current study was to evaluate the effect of the scaling up of this QI package on intrapartum-related mortality and neonatal resuscitation care.

## Methods

This study is reported as per the Consolidated Standards of Reporting Trials (CONSORT) guideline (S1 CONSORT Checklist).

### Study design

**Fig 1:**
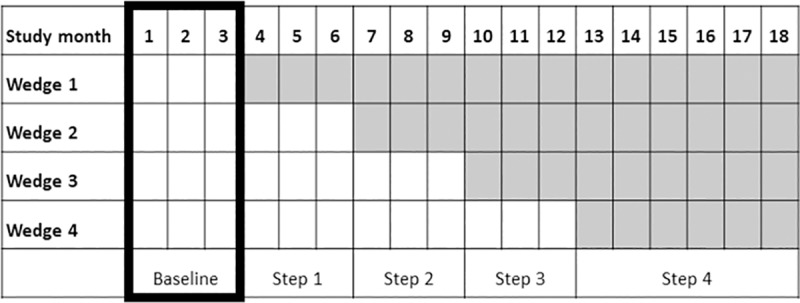
Stepped-wedge design and time line of the NePeriQIP trial. The period within bold lines represents the baseline period. The shaded period constitutes the intervention period. Each wedge contains three hospitals (clusters). NePeriQIP, Nepal Perinatal Quality Improvement Package.

A stepped-wedge cluster randomized controlled design was applied, with 12 public hospitals in Nepal as the clusters [34]. The study period followed the Nepali calendar and was initiated on April 14, 2017 (2073/1/1), and ran for 18 months up until October 17, 2018 (2075/6/31). Thus, according to published study protocol, the QI package was introduced at three hospitals at the same time with a three-month interval between each wedge [34]. Four of the hospitals were high-volume (>8,000 deliveries a year), four medium-volume (>3,000 deliveries a year), and the remaining four low-volume (>1,000 deliveries a year) hospitals, and each wedge included a hospital from these three categories. Each cluster had a control and an intervention period. The first three months of the study constituted a baseline period, when no intervention activities took place at any of the 12 hospitals (Fig 1).

### Study setting

Hospitals were selected based on the criteria of having more than 1,000 deliveries per year and a referral center for maternal and newborn care. All the hospitals provided normal vaginal, assisted vaginal, and cesarean section delivery series. The small-sized hospitals (Bardiya, Pyuthan, Nuwakot, and Nawalparasi) did not have specialized sick newborn care service. The high-volume hospitals (Koshi Zonal, Bharatpur, Lumbini Zonal, and Bheri Zonal) provided specialized sick newborn care service. The medium-volume hospitals (Western Regional, Rapti Sub-Regional, Mid-Western Regional, and Seti Zonal) provided specialized newborn care services. All the hospitals, despite mostly being in the flatlands, were different in terms of service coverage and diverse in relation to ethnicity, language, and religion. Mid-Western Regional and Seti Zonal Hospitals were located in the most disadvantaged regions in Nepal in terms of literacy, access to services, and life expectancy. Bheri Zonal Hospital in Nepalgunj had a large number of minority (Muslim) communities, while in Bharatpur, some of the most fringe communities come for maternal and sick newborn services. The hospitals vary in terms of service delivery to ethnic minorities, population age groups, and cultural practices. The labor unit in each hospital was led by skilled birth attendants and had access to neonatal resuscitation services at birth. Pediatricians led these neonatal care units. In the low-volume hospitals, sick newborns were managed at the pediatric unit, which was led by medical doctors. During the baseline period, the intrapartum-related mortality rate ranged from 9 to 31 per 1,000 births at the 12 hospitals, with a mean of 13 per 1,000 births.

### Participants

Women at 22 weeks of gestation or more admitted in the labor room, whose fetal heart sound was heard at the time of admission, were eligible for inclusion. Any women who had antepartum stillbirth or no fetal heart sound at admission were excluded.

### Intervention

At each hospital, the QI package was introduced in a similar manner in the following steps. Firstly, an orientation of the hospital management committee was arranged in order to strengthen leadership and accountability for perinatal care (meso level). Together with the management, an assessment of service readiness and availability of intrapartum care, as well as a bottleneck analysis, was conducted. Based on the findings, a plan for improvement of quality of care was developed. Secondly, in-hospital facilitators were appointed by the management and trained to lead the QI process and facilitate delivery staff (meso- and microlevel) [30]. This was done through capacity building of the components of the QI process and based on the PDSA methodology to be used at biweekly peer review meetings [30]. Thirdly, health staff were introduced to the QI process, which consisted of (1) an introduction of and training in neonatal resuscitation standards and Essential Newborn Care (ENC) and (2) daily drills to practice neonatal resuscitation skills on a mannequin. Finally, a quality metric score card for the quality of neonatal resuscitation and outcome of care was established (microlevel). Training neonatal resuscitation and ENC was carried out by trainers who received training from trainers from the team from the American Academy of Pediatrics on basic neonatal resuscitation, Helping Babies Breathe (HBB 1.0) (S2 Text) [35].

### Outcome variables

#### Primary outcome

- Intrapartum-related mortality defined as intrapartum stillbirth (no breathing 10 minutes after delivery) and neonatal death within the first 24 hours of life.

#### Secondary outcome

- Bag-and-mask ventilation, stimulation, and suctioning performed on nonbreathing newborns.
- Early neonatal deaths (i.e., first seven-day neonatal mortality).

### Power calculation

Power calculations based on an estimated primary outcome level of 20/1,000 births and an annual delivery rate of 60,000 at the 12 hospitals combined would allow us to demonstrate a significant reduction of intrapartum-related mortality of 14/1,000 births or more (alfa 0.05, beta 0.80). Power calculations were performed with R package cluster randomized trial (CRT)size. Reductions in intrapartum-related mortality at each of the 12 hospitals followed a before-after design, with variable length of control and intervention periods depending on the allocation to different wedges.

### Randomization

Using block randomization, the high-, medium-, and low-volume hospitals were randomly allocated into one of the wedges. The last author generated the random sequence. Because this is implementation research, the allocation of treatment/intervention could not be concealed. The research and implementation team knew the outcome expected from the study. The randomization sequence was generated prior to the study start. The hospital did not have prior knowledge of when the intervention would be rolled out. Information on the rolling out of the intervention in the hospital was provided a month ahead of rollout.

The data collection and management team was not aware of when the hospital was in a control or intervention period. The independent data collection and management team was not engaged in the planning and implementation of the intervention.

### Data collection and management

For the collection of data on primary and secondary outcomes, an independent data collection team was established at each hospital. In all hospitals, the mortality outcomes were collected from the women’s case notes, including the clinical events (mode of delivery, birth weight, gestational age, and neonatal resuscitation practices at birth). The demographic characteristics of the women (age, ethnicity, parity, and education) were collected through semi-structured exit interviews upon discharge. The data collection was carried out in a paper-based format. Following the completion of the forms, data collectors at each site sent the completed forms in a sealed envelope weekly via courier to the central research office in Kathmandu. At the central research office, the forms were rechecked for completeness and were entered into a Census and Survey Processing System (CS-Pro) database. All the entered forms were indexed according to respective hospital. The data cleaning of the data in the CS-Pro was done on a monthly basis. A standard operating protocol (SOP) was developed for data collection and management (S3 Text). A quarterly data quality assurance assessment was conducted by the central level investigation team to assess adherence to the SOP. Feedback was provided to the data collection team on adherence to the SOP.

### Data analysis

Descriptive analyses of the demographic, obstetric, and neonatal characteristics of the population groups in control and intervention periods were performed to assess cluster variation. We calculated the proportion and CI of the characteristics of the populations in control and intervention periods using the STATA I/C 12.1. Using the software R (version 3.4.0), we analyzed change in mortality outcomes between the intervention and control periods by generalized linear mixed model (GLMM). Analyses were performed with R package lme4. Intra-cluster correlation coefficients (ICCs) were calculated for each outcome. For the subgroup analysis on the intrapartum-related mortality incidence change by wedge and size of hospitals during control and intervention period treatments, Pearson chi-squared test and binary logistic regression were used. Forward modeling was applied, adding obstetric and infant characteristic variables one at a time using Statistical Package for the Social Sciences (SPSS) 25.0. If the odds ratio (OR) was affected by 10% in either direction, the variable was maintained for the wedge and size of the hospital. Missing values in each variable were excluded from analysis. All analyses were carried out as per intention to treat (ITT).

### Ethical considerations

Ethical approval was obtained from the Nepal Health Research Council (ref 26–2017). The delay in obtaining the approval of the QI package document led to a delay in clinical trial registration. The stepped-wedge design allowed all included hospitals to receive outcome information regarding the intervention, which has previously been proven beneficial. Written Informed consent was obtained from women upon admission and before the exit interview.

## Results

**Fig 2:**
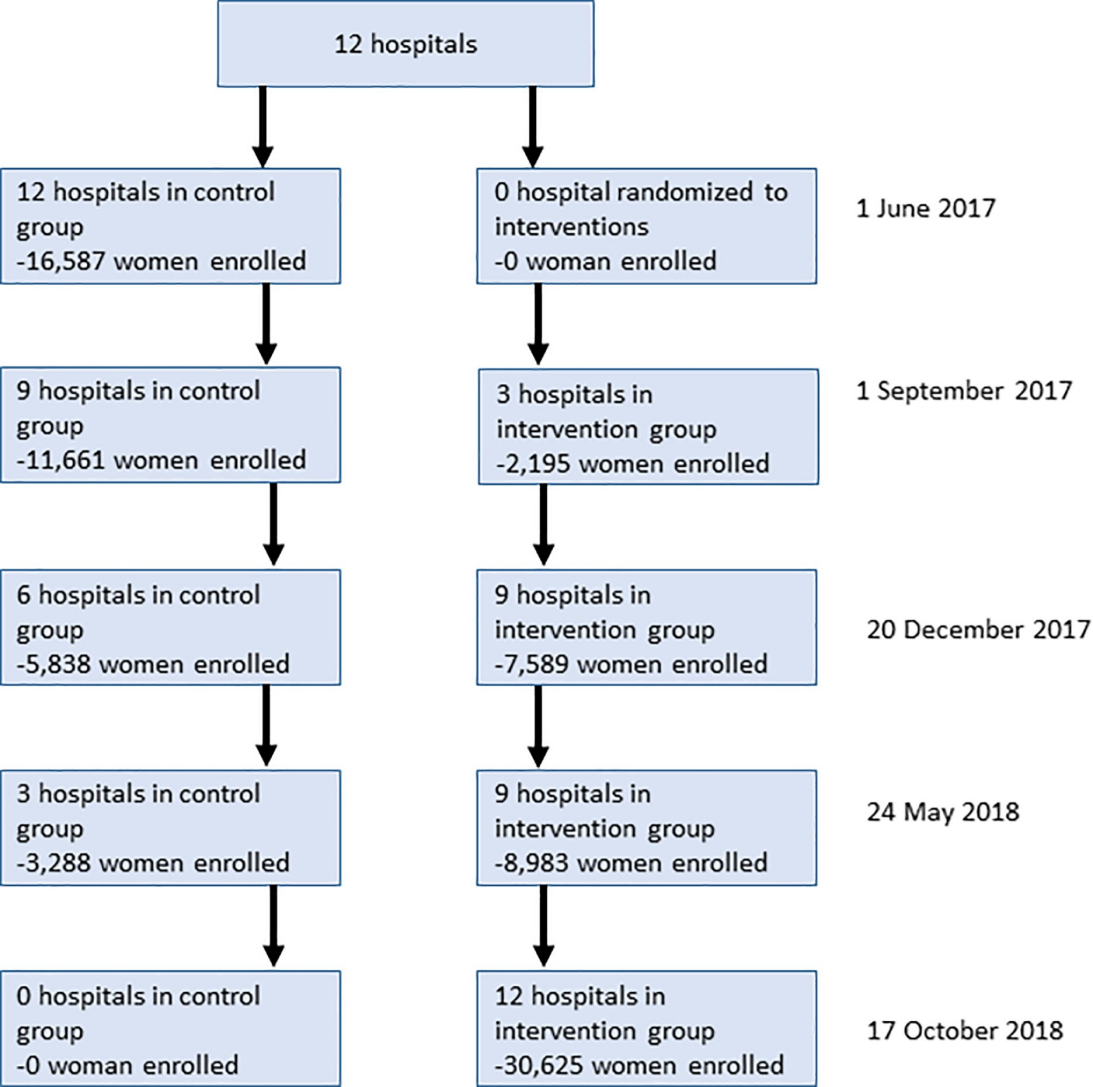
CONSORT trial profile.

A total of 92,322 women were eligible to be included in the study in the primary ITT analysis. Of these, 3,808 women were excluded, as they were referred to other facilities for delivery or declined to participate in the study. This resulted in 88,524 deliveries being recorded during the 18-month study period (Fig 2).

Of these, 490 (0.6%) women had multiple births; therefore, 89,014 infants were included in the final analysis. The overall intrapartum-related mortality rate was 9.1/1,000 deliveries (Table 1).

**Table 1:** The total delivery, live births, and mortality rate during the study period.

Outcome	*n*	Rate (CI 95%)
Deliveries	88,524	-
Live births	88,515	-
		
Multiple deliveries	490	55/1,000 (50.4–60.3)
Cesarean sections	18,296	206/1,000 (204–209)
		
Intrapartum stillbirths	499	5.6/1,000 (5.1–6.1)
First day mortality	307	3.5/1,000 (3.1–3.9)
Early neonatal mortality	990	11.2/1,000 (10.5–11.9)

The mean age of the mother in the study period was 24.0 ± 4.3 years, with 54.9% (95% CI, 54.6–55.2) from disadvantaged ethnic groups and 4.0% (95% CI, 3.9–4.1) illiterate. Among the study participants, 45.4% (95% CI, 45.0–45.8) were first time mothers, 3.2% (95% CI, 3.1–3.3) had complication during labor, and 14.9% (95% CI, 14.7–15.1) had emergency cesarean section. Of the total births, 54.4% (95% CI, 54.0–54.8) were boys, 16.7% (95% CI, 16.5–16.9) had gestational age less than 37 weeks, and 17.1% (95% CI, 16.9–17.3) had birth weight less than 2,500 grams (Table 2).

**Table 2:** Demographic, obstetric, and neonatal characteristics of the population in the control and intervention periods as ITT.

Maternal-neonatal characteristics	Control (*N* = 38,378, % with CI)	Intervention (*N* = 50,636, % with CI)	Total (*N* = 89,014, % with CI)
Maternal age (mean ± SD)	23.9 ± 4.3	24.1 ± 4.4	24.0 ± 4.3
Disadvantaged ethnic group	56.2% (54.7–56.7)	56.5% (56.1–56.9)	54.9% (54.6–55.2)
Illiterate	4.1% (3.9–4.3)	3.9% (3.7–4.1)	4.0% (3.9–4.1)
Parity			
Nullipara	49.0% (48.5–49.5)	45.0% (44.6–45.4)	45.4% (45.0–45.8)
Primipara	34.9% (34.5–35.4)	35.3% (34.9–35.7)	35.1% (34.7–35.5)
Multipara	19.7% (19.3–20.1)	19.2% (18.9–19.5)	19.4% (19.1–19.7)
Sex of baby			
Boy	53.5% (53.0–54.0)	54.4% (54.0–54.8)	45.4% (45.0–45.8)
Girl	46.5% (46.0–47.0)	45.6% (45.2–46.0)	35.1% (34.7–35.5)
Mode of delivery*			
Normal vaginal	76.1% (75.7–76.5)	73.2% (72.8–73.6)	74.4% (74.1–74.7)
Assisted vaginal	3.9% (3.7–4.1)	4.0% (3.8–4.2)	3.9% (3.8–4.0)
Emergency cesarean section	13.9% (13.6–14.2)	15.6% (15.3–15.9)	14.9% (14.7–15.1)
Elective cesarean section	5.3% (5.1–5.5)	6.5% (6.3–6.7)	6.0% (5.8–6.2)
Not recorded	0.9% (0.8–1.0)	0.7 (0.6–0.6)	0.8 (0.7–0.9)
			
Complication during labor	3.0% (2.8–3.2)	3.4% (3.2–3.6)	3.2% (3.1–3.3)
			
Gestational age (mean ± SD)	38.1 ± 2.6	38.2 ± 2.6	38.1 ± 2.6
Gestational age categorized**			
37 weeks and more	82.8% (82.4–83.2)	83.7% (83.4–84.0)	83.3% (83.1–83.5)
Less than 37 weeks	17.2% (16.8–17.6)	16.3% (16.0–16.6)	16.7% (16.5–16.9)
			
Birth weight (mean ± SD)	2,795.9 ± 478.3	2,785.9 ± 469.2	2,794.7 ± 478.8
Birth weight categorized***			
2,500 grams and more	82.7% (82.3–83.1)	83.1% (82.8–83.4)	82.9% (82.7–83.1)
Less than 2,500 grams	17.3% (16.9–17.7)	16.9% (16.6–17.2)	17.1% (16.9–17.3)

*Missing: 1,273.

**Missing: 1,001.

***Missing: 1,029.

Abbreviation: ITT, intention to treat.

Intervention start at the various hospitals deviated from the intended dates due to external factors and unforeseen circumstances. All analyses were, however, conducted as per ITT (S1 Table).

Based on the ITT, using GLMM analyses, the incidence of intrapartum-related mortality was 10.7 per 1,000 births during the control period and 7.8 per 1,000 births during the intervention period (aOR, 0.79; 95% CI, 0.69–0.92; *p* = 0.002; ICC, 0.0286). The incidence of intrapartum stillbirth was 6.8 per 1,000 births during the control period and 4.7 per 1,000 births during the intervention period (aOR, 0.73; 95% CI, 0.61–0.88; *p* < 0.001; ICC, 0.1160). The incidence of first day neonatal mortality was 3.9 per 1,000 live births during the control period and 3.1 per 1,000 live births during the intervention period (aOR, 0.92; 95% CI, 0.73–1.16; *p* = 0.49; ICC, 0.1557). The incidence of early neonatal mortality was 12.7 per 1,000 live births during the control period and 10.1 per 1,000 live births during the intervention period (aOR, 0.89; 95% CI, 0.78–1.02, *p* = 0.09; ICC, 0.1538) (Table 3).

**Table 3:** Mortality rate in control and intervention periods (ITT) using GLMM.

Mortality rate	Control period		Intervention period		aOR	CI 95%	*p*-value	ICC
	Deaths/births	Rate per 1,000 births (95% CI)	Deaths/births	Rate per 1,000 births (95% CI)				
Intrapartum-related mortality	409/38,378	10.7 (9.6–11.7)	397/50,636	7.8 (7.1–8.6)	0.79	0.69–0.92	0.002	0.0286
Intrapartum stillbirth	260/38,378	6.8 (6.0–7.6)	239/50,636	4.7 (4.1–5.3)	0.73	0.61–0.88	<0.001	0.1160
First day mortality	149/38,118	3.9 (3.3–4.5)	158/50,397	3.1 (2.6–3.6)	0.92	0.73.–1.16	0.49	0.1557
Early neonatal mortality	482/38,118	12.7 (10.9–13.1)	508/50,397	10.1 (9.2–11.0)	0.89	0.78–1.02	0.09	0.1538

Abbreviations: aOR, adjusted odds ratio; GLMM, generalized linear mixed model; ICC, intra-cluster correlation coefficient; ITT, intention to treat.

In the subgroup analysis, wedge 2 hospitals had an incidence rate of 12.5 per 1,000 births in the control period and 7.0 per 1,000 births in the intervention period (aOR, 0.70; 95% CI, 0.50–0.99; *p* = 0.044). Among the size of the hospital, small-sized hospitals had incidence of intrapartum-related deaths from 15.2 per 1,000 births in the control period to 6.7 per 1,000 births in the intervention period (aOR, 0.43; 95% CI, 0.26–0.72; *p* = 0.002). Among the individual hospitals, the 10th hospital (Lumbini) had an incidence rate of 14.2 per 1,000 births in the control period to 11.3 per 1,000 births in the intervention period (aOR, 0.72; 95% CI, 0.52–0.99; *p* = 0.017). Among the individual hospitals, the 11th hospital had an incidence rate of 7.9 per 1,000 births in the control period and 15.7 per 1,000 births in the intervention period (aOR, 1.98; 95% CI, 1.28–3.07; *p* = 0.002) (Table 4).

**Table 4:** Intrapartum-related mortality rates (deaths/births) in control and intervention periods by wedge, hospital size, and individual hospitals (ITT).

Site	Deaths/births	Rate per 1,000 births (95% CI)	Deaths/births	Rate per 1,000 births (95% CI)	cOR (95% CI)	aOR (95% CI)^1^	*p*-value
Wedge 1	55/4,414	12.5 (9.4–16.1)	93/13,299	7.0 (5.6–8.6)	0.73 (0.64–0.84)	0.70 (0.50–0.99)^2^	0.044
Wedge 2	92/11,891	7.7 (6.2–9.5)	98/19,168	5.1 (4.2–6.2)	0.66 (0.50–0.88)	0.69 (0.52–0.93)	0.012
Wedge 3	86/8,028	10.7 (8.6–13.2)	82/8,748	9.4 (7.5–11.6)	0.87 (0.65–1.18)	0.91 (0.67–1.23)	0.411
Wedge 4	176/14,045	12.5 (10.8–14.5)	124/9,421	13.2 (101.0–15.7)	1.05 (0.83–1.33)	0.93 (0.74–1.18)^2^	0.569
Volume hospital	Control	Rate	Intervention	Rate	cOR	aOR	*p*-value
High volume	252/22,348	11.3 (9.9–12.7)	222/29,368	7.6 (6.6–8.6)	0.67 (0.56–0.80)	0.69 (0.57–0.82)	<0.001
medium volume	107/12,732	8.4 (6.9–10.1)	153/17,938	8.5 (7.2–10.0)	1.02 (0.79–1.30)	1.06 (0.83–1.36)	0.796
low volume	50/3,298	15.2 (11.3–19.9)	22/3,330	6.7 (4.2–9.9)	0.43 (0.26–0.72)	0.46 (0.27–0.76)	0.002
By hospital	Control	Rate	Intervention	Rate	cOR	aOR	*p*-value
Hospital 1	35/3,070	11.4 (8.0–15.8)	52/7,658	6.8 (5.1–8.9)	0.59 (0.39–0.91)	0.74 (0.48–1.14)	0.160
Hospital 2	17/1,128	15.1 (8.8–224.0)	39/4,715	8.3 (5.9–11.3)	0.55 (0.31–0.97)	0.69 (0.38–1.25)	0.217
Hospital 3	3/216	13.9 (2.9–40.1)	2/926	2.2 (0.3–7.8)	0.15 (0.03–0.93)	0.22 (0.03–1.41)	0.069
Hospital 4	59/6,864	8.6 (6.5–11.1)	68/11,155	6.1 (4.7–7.7)	0.71 (0.50–1.00)	0.72 (0.51–1.02)	0.081
Hospital 5	25/4,272	5.9 (3.8–8.6)	24/6,843	3.5 (2.2–5.2)	0.60 (0.34–1.05)	0.66 (0.37–1.16)	0.092
Hospital 6	8/755	10.5 (5.6–20.8)	6/1,170	5.1 (1.9–11.1)	0.48 (0.17–1.39)	0.59 (0.20–1.78)	0.390
Hospital 7	49/4,757	10.3 (7.6–13.6)	39/4,968	7.9 (5.6–10.7)	0.76 (0.50–1.16)	0.75 (0.49–1.15)	0.168
Hospital 8	28/2,631	10.6 (7.4–15.4)	43/3,380	12.7 (9.4–17.1)	1.20 (0.74–1.93)	1.30 (0.79–2.14)	0.457
Hospital 9	9/640	14.1 (4.1–26.9)	0/400	0.0 (0.0–0.0)	–	–	-
Hospital 10	109/7,657	14.2 (11.7–17.1)	63/5,587	11.3 (8.7–14.4)	0.79 (0.58–1.08)	0.72 (0.52–0.99)	0.017
Hospital 11	37/4,701	7.9 (5.5–10.8)	47/3,000	15.7 (11.5–20.8)	2.01 (1.30–3.10)	1.98 (1.28–3.07)	0.002
Hospital 12	30/1,687	17.8 (12.0–25.3)	14/834	16.8 (9.2–28.0)	0.94 (0.50–1.79)	0.84 (0.44–1.62)	0.554

^1^Forward modelling test for mode of delivery, preterm birth, and sex of baby.

^2^Adjusted for preterm birth.

Abbreviations: aOR, adjusted odds ratio; cOR, crude odds ratio; ITT, intention to treat.

**Fig 3:**
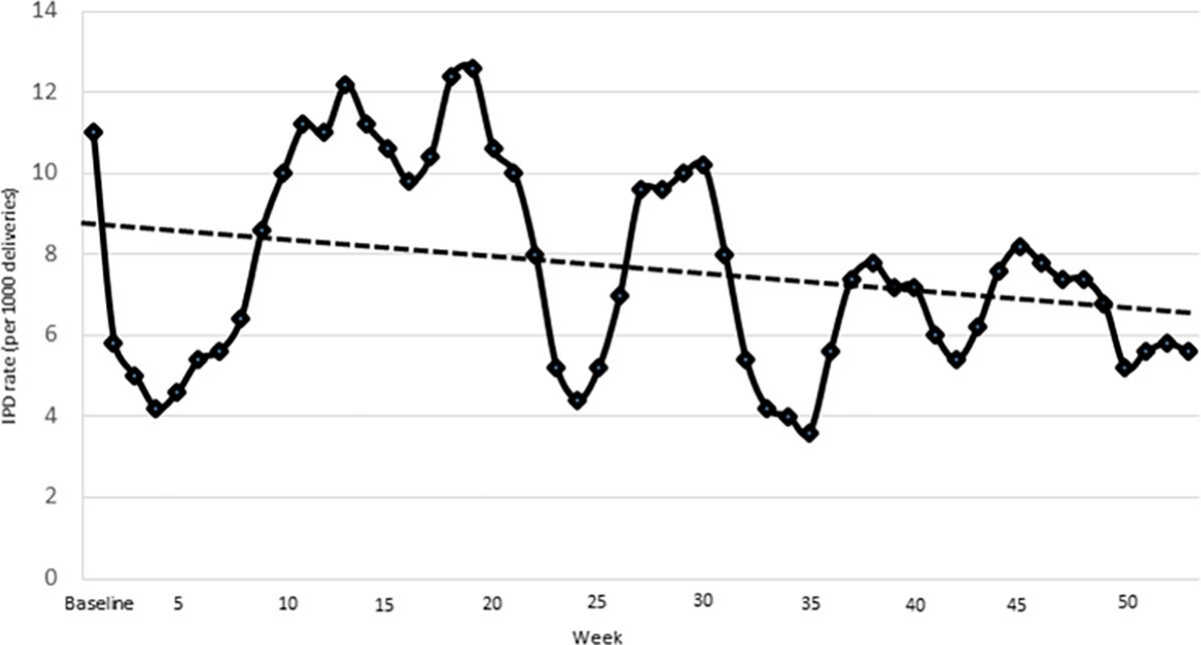
Trend in intrapartum related mortality rate (per 1,000 births).

Analysis of the intrapartum mortality rate on a weekly basis after the start of the intervention over the 52 weeks following intervention showed produced a downward trend (Fig 3). In this visualization of the intervention effect, the first two wedges contribute to 52 weeks, the third wedge contributes 40 weeks, and the last wedge 28 weeks, resulting in a declining overall sample.

**Fig 4:**
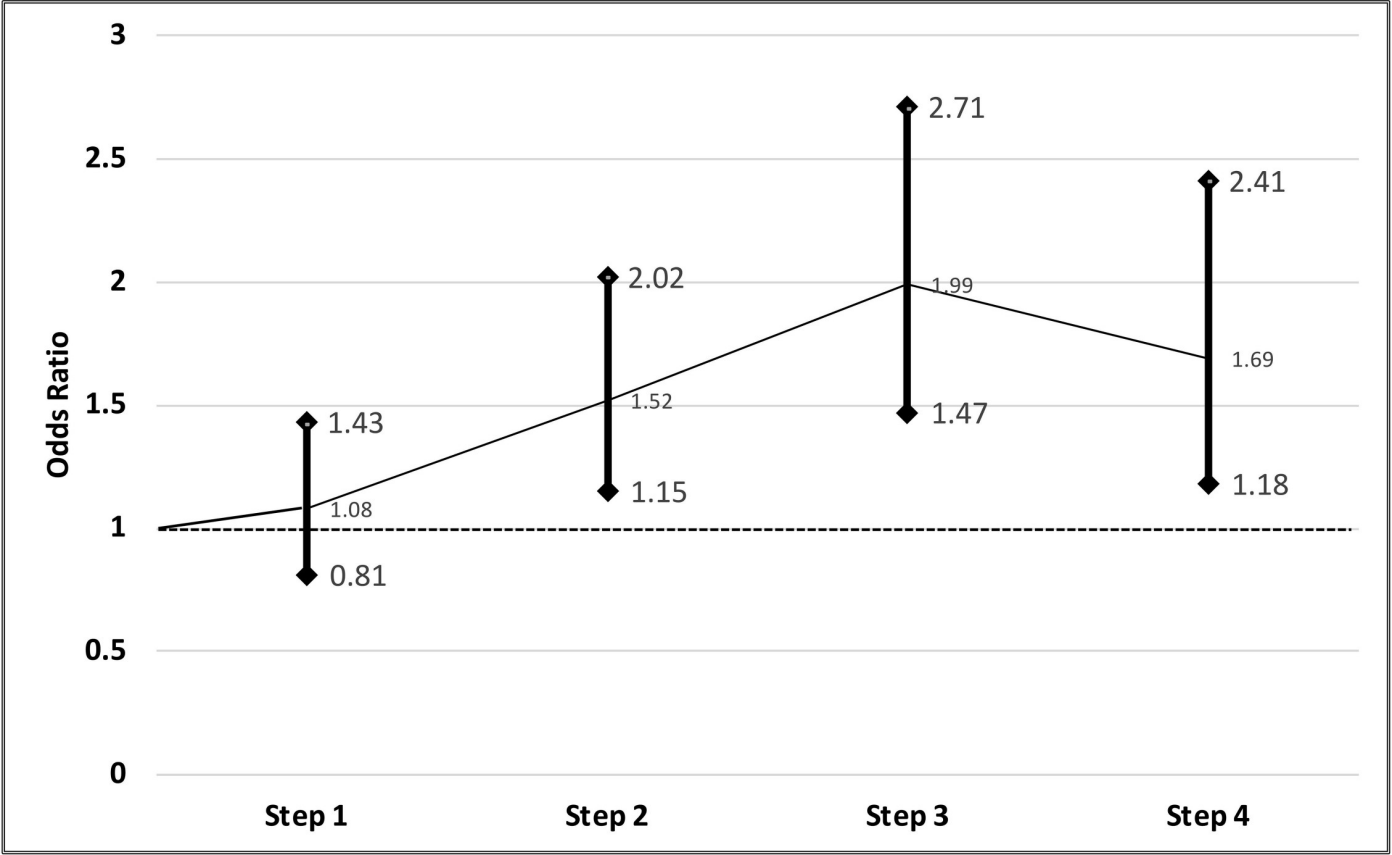
aORs (GLMM) for bag-and-mask resuscitation during the four steps of implementation (baseline period as reference). aOR, adjusted odds ratio; GLMM, generalized linear mixed model.

The use of bag-and-mask ventilation for babies with low Apgar score (<7 at 1 minute) increased from 3.2% in the control period to 4.0% by 52% in the intervention period (aOR, 1.52; 95% CI, 1.32–1.77; *p* = 0.003). During baseline, bag-and-mask ventilation was performed on 0.6% (104/16,699) of all newborns and on 2.5% (100/3,993) of all newborns with an Apgar score of <7 at 1 minute after birth. During the first three months after the actual intervention had started, bag-and-mask ventilation had increased to 1.3% (201/15,290) (*p* < 0.001) and to 5.4% (172/3,211) (*p* < 0.001) for infants with Apgar <7 at 1 minute. This increased likelihood of an infant receiving bag and mask after intervention remained in the overall sample even after adjusting for cluster (aOR, 1.52; 95% CI, 1.32–1.77; *p* < 0.001). Not only did the likelihood of receiving bag-and-mask ventilation increase after intervention, but also resuscitation by drying and stimulation (aOR, 2.99; 95% CI, 2.87–3.11; *p* < 0.001) as well as suctioning (aOR, 2.28; 95% CI, 2.10–2.49; *p* < 0.001). Infants were most likely to receive bag-and-mask ventilation during the third step of the study period, when newborns were twice as likely to receive ventilation with bag and mask compared with during baseline (aOR, 1.99; 95% CI, 1.47–2.71; *p* < 0.001) (Fig 4).

## Discussion

Results indicate that scaling up a package of QI interventions for improved intrapartum survival is feasible and also has the potential to increase the quality of care in real-life settings. We observed effects in clinical practice, as bag-and-mask ventilation for babies and suctioning increased. However, not all hospitals included in the trial displayed the same process and results, testifying to the diversity of contexts and conditions of scaled-up efforts. Further analyses to understand the contextual implications are needed.

We believe that this study will add to the global and national discussion about the effectiveness of meso- and microlevel QI interventions in bringing changes in quality of care [16, 36]. The “Lancet Commission for High Quality Health System” 2018 report recommends investing in QI initiatives at macro-level, suggesting the setting up of quality governance units at the central Ministry of Health and Population [10]. However, there is lack of evidence from the recent systematic review that macro-level QI intervention affects the quality of care [15]. This is more a utopian call to countries like Nepal to have QI interventions delivered at levels simultaneously, where the centralized governance system has dwindled into a federal system. In such states, improving the meso-level environment for introducing change remains to be the option for implementing change.

One of the large-scale cluster randomized controlled trials on the effect of the implementation of WHO’s Safer Birth Checklist on quality of care and birth outcome, using external facilitators in a healthcare facility in India, showed improvement in the intrapartum care in the intervention area but did not show changes in maternal and perinatal mortality [37]. In a cluster-randomized trial in 12 rural primary care centers in Tanzania, a QI intervention (training, mentoring, infrastructure support, and peer outreach) improved quality antenatal care [38]. A multicountry observational study showed that rapid scale-up of training of neonatal resuscitation was associated with improvement in overall neonatal resuscitation practice [39] and improvement in perinatal mortality in a subset of the population [40]. In line with these findings, our study reports that implementation of a QI package (facilitation, training, weekly meeting, and outreach visit) using facilitators improved resuscitation practice and reduced intrapartum-related mortality. We have demonstrated that, if health institution leaders engaged in implementing QI processes and made it a common vision to implement change, improvements in survival can take place. Coupled with improved governance for care, introducing QI processes such as facilitated PDSA cycles and facilitation will help implement change at the clinical units. The quality metric system on key neonatal resuscitation care established in each labor unit is an accountability trigger for better governance of care.

While undertaking the review process to develop the QI package through multi-stakeholder consultation, we found a lack of QI packages for intervention at different levels of governance to improve the quality of intrapartum care. Therefore, we developed the QI package so that it would intervene at both meso- (hospital and clinical unit leaders) and micro- (individual health workers) levels. Nor did we find any randomized controlled trials that assessed the effectiveness of QI interventions on neonatal resuscitation care and intrapartum-related mortality.

### Methodological considerations

The study design, stepped-wedge cluster randomized trial, has many advantages. By mimicking a classical randomized control trial (RCT), this pragmatic design allowed all clusters to be exposed to the intervention by the end of the study. At the beginning of the study, there will be an initial period with no cluster exposed to intervention. At a three-month interval, one cluster was randomized to cross from control to intervention. At the end of the study, for a period all clusters were exposed to the intervention. [41,42,43]. The study design also allows for the practical implementation of the intervention that would have been very resource demanding if it had been carried out at the same time at all hospitals.

NePeriQIP was a hospital-based study in district and regional hospitals to improve the quality of neonatal resuscitation care. The selection of hospitals was representative of different levels of the public health system of the country. The range of annual delivery, human resources, infrastructure, and equipment strengthened the generalizability of results, both for similar settings in Nepal as well as in other low- and middle-income settings.

Another strength of the current study is the large sample size and the diverse settings it covers. Most importantly, by combining intrapartum stillbirths and first day mortality into the primary outcome of intrapartum-related mortality, we reduced the risk of misclassification. Early neonatal mortality risk was observed to be 11% lower in the intervention period than in the control period, but there was only moderate statistical evidence to conclude that the risk was truly different between the time periods.

There are, however, several limitations to the study that are worth mentioning. Although a large sample size of almost 90,000 women–infant pairs were enrolled in the study, the clustering reduces the power of the study. Consequently, we did not detect a reduction in first day mortality, as it was not sufficiently powered to detect the minimal change with the number of clusters provided. The delay in the start of intervention could have biased the results; however, intrapartum-related mortality subgroup analysis as per the protocol showed similar results (S1 Table, S2 Table, S3 Table). A stepped-wedge design involving clusters joining in a predetermined sequence, with each preceding cluster as intervention and the successive cluster as control, is subject to a threat of internal validity [44]. The successive clusters were exposed to the diffusion of treatment effect, as the intervention was widely disseminated in the national forum and the single hospital–based QI study had received widespread attention. Hospitals in successive clusters may also have engaged in competitive behavior and increased the effort to improve the resuscitation practice while waiting to start the intervention. Stepped-wedge trials (SWTs) randomly allocate clusters to control groups that cross over the intervention at different crossover points. The secular trend in the mortality outcome may impact the reporting of the outcome [45]. We reported the secular trend in Fig 2. To adjust the secular trend of mortality, time trends were entered into the model as fixed effects in GLMM, with the assumption that the trend was similar in all clusters.

The analysis for our main outcome is based on ITT. The intervention started at most hospitals, however, varied due to societal and organizational factors. At one hospital, there was a long and unforeseen strike, and factors like this paired with logistical considerations made it difficult to start at the same—and intended—dates of each wedge. These unforeseen events are, however, the nature of implementation research, and most likely, the effect of the intervention would have increased if all hospitals had received the intervention when intended. Finally, this was a large-scale trial recording a large number of deliveries over a relatively short period. A rigorous follow-up and control of data completeness were carried out continuously to minimize data loss. Data implausibility was detected at a late stage in relation to birth weights and gestational age at three hospitals. Data on these variables were thus discarded from these hospitals.

This study demonstrates a reduction in intrapartum-related mortality after implementation of a scaled-up QI intervention for neonatal resuscitation in public hospitals in Nepal. Using the learning from the implementation of the QI package, implementation of the package in other settings can have impact on intrapartum-related mortality. Further assessments of the sustainability of the QI process and improved quality of care in the hospital and labor units are a priority area of research.

## Supporting information

S1 CONSORT Checklist (PDF)

S1 Text National consultation for developing the QI guideline. QI, quality improvement.
(PDF)

S2 Text QI guideline. QI, quality improvement.
(PDF)

S3 Text Collection and management of the SOP. SOP, standard operating protocol.
(PDF)

S1 Table Time line of intervention in each hospital. (PDF)

S2 Table Intrapartum-related mortality GLMM analysis per protocol. GLMM, generalized linear mixed model.
(PDF)

S3 Table Intrapartum-related mortality subgroup analysis per protocol. (PDF)

## Abbreviations

aOR: adjusted odds ratio
CRT: cluster randomized trial
CS-Pro: Census and Survey Processing System
ENC: Essential Newborn Care
GLMM: generalized linear mixed model
HBB: Helping Babies Breathe
ICC: intra-cluster correlation coefficient
ITT: intention to treat
NePeriQIP: Nepal Perinatal Quality Improvement Package
OR: odds ratio
PDSA: Plan-Do-Study-Act
QI: quality improvement
RCT: randomized control trial
SDG: Sustainable Development Goal
SOP: standard operating protocol
SPSS: Statistical Package for the Social Sciences
SWT: stepped-wedge trial
