# Supplementary material for: Effect of a scaled-up neonatal resuscitation quality improvement package on intrapartum-related mortality in Nepal: A stepped-wedge cluster randomized controlled trial
Source: PLoS Med. 2019 Sep 9;16(9):e1002900. doi: 10.1371/journal.pmed.1002900 (PMC6733443; doi:10.1371/journal.pmed.1002900)
Supplement: S1 CONSORT Checklist — (PDF) [file pmed.1002900.s001.pdf]

S2. CONSORT checklist

Section/Topic	Item No	Checklist item	Reported on section and paragraph #
**Title and abstract**			
	1a	Identification as a randomised trial in the title	1st paragraph
	1b	Structured summary of trial design, methods, results, and conclusions (for specific guidance see CONSORT for abstracts)	Abstract section, paragraphs 1-5
**Introduction**			
Background and objectives	2a	Scientific background and explanation of rationale	Introduction section, paragraphs 1-4
	2b	Specific objectives or hypotheses	Introduction section, paragraph 5
**Methods**			
Trial design	3a	Description of trial design (such as parallel, factorial) including allocation ratio	Method section, paragraph 2
	3b	Important changes to methods after trial commencement (such as eligibility criteria), with reasons	
Participants	4a	Eligibility criteria for participants	Method section, paragraph 4
	4b	Settings and locations where the data were collected	Method section, paragraph 3
Interventions	5	The interventions for each group with sufficient details to allow replication, including how and when they were actually administered	Method section, paragraph 5
Outcomes	6a	Completely defined pre-specified primary and secondary outcome measures, including how and when they were assessed	Method section, paragraph 6 and 7
	6b	Any changes to trial outcomes after the trial commenced, with reasons	
Sample size	7a	How sample size was determined	Method section, paragraph 8
	7b	When applicable, explanation of any interim analyses and stopping guidelines	
Randomisation:			
Sequence generation	8a	Method used to generate the random allocation sequence	Method section, paragraph 9

	8b	Type of randomisation; details of any restriction (such as blocking and block size)	Method section, paragraph 10
Allocation concealment mechanism	9	Mechanism used to implement the random allocation sequence (such as sequentially numbered containers), describing any steps taken to conceal the sequence until interventions were assigned	Method section, paragraph 10
Implementation	10	Who generated the random allocation sequence, who enrolled participants, and who assigned participants to interventions	Method section, paragraph 10
Blinding	11a	If done, who was blinded after assignment to interventions (for example, participants, care providers, those assessing outcomes) and how	Method section, paragraph 10-11
	11b	If relevant, description of the similarity of interventions	Discussion section, paragraph 3
Statistical methods	12a	Statistical methods used to compare groups for primary and secondary outcomes	Method section, paragraph 11-12
	12b	Methods for additional analyses, such as subgroup analyses and adjusted analyses	Method section, paragraph 12
**Results**			
Participant flow (a diagram is strongly recommended)	13a	For each group, the numbers of participants who were randomly assigned, received intended treatment, and were analysed for the primary outcome	Results section, paragraph 1
	13b	For each group, losses and exclusions after randomisation, together with reasons	Results section, paragraph 1
Recruitment	14a	Dates defining the periods of recruitment and follow-up	Results section, paragraph 3
	14b	Why the trial ended or was stopped	
Baseline data	15	A table showing baseline demographic and clinical characteristics for each group	Results section, paragraph 2, table 1 and 2
Numbers analysed	16	For each group, number of participants (denominator) included in each analysis and whether the analysis was by original assigned groups	Results section, paragraph 4
Outcomes and estimation	17a	For each primary and secondary outcome, results for each group, and the estimated effect size and its precision (such as 95% confidence interval)	Results section, paragraph 4
	17b	For binary outcomes, presentation of both absolute and relative effect sizes is recommended	Results section, table 3
Ancillary analyses	18	Results of any other analyses performed, including subgroup analyses and adjusted analyses, distinguishing pre-specified from exploratory	Results section, paragraph 5-6, table 4

Harms	19	All important harms or unintended effects in each group (for specific guidance see CONSORT for harms)	
**Discussion**			
Limitations	20	Trial limitations, addressing sources of potential bias, imprecision, and, if relevant, multiplicity of analyses	Discussion section, paragraph 6
Generalisability	21	Generalisability (external validity, applicability) of the trial findings	Discussion section, paragraph 2-5
Interpretation	22	Interpretation consistent with results, balancing benefits and harms, and considering other relevant evidence	Discussion section paragraph 1-2 and 7 conclusion section, paragraph 1
**Other information**			
Registration	23	Registration number and name of trial registry	Title page, paragraph 2
Protocol	24	Where the full trial protocol can be accessed, if available	Title page, paragraph 2
Funding	25	Sources of funding and other support (such as supply of drugs), role of funders	Footnote, paragraph 4

*We strongly recommend reading this statement in conjunction with the CONSORT 2010 Explanation and Elaboration for important clarifications on all the items. If relevant, we also recommend reading CONSORT extensions for cluster randomised trials, non-inferiority and equivalence trials, non-pharmacological treatments, herbal interventions, and pragmatic trials. Additional extensions are forthcoming: for those and for up to date references relevant to this checklist, see www.consort-statement.org.
