# Supplementary material for: Effect of a scaled-up neonatal resuscitation quality improvement package on intrapartum-related mortality in Nepal: A stepped-wedge cluster randomized controlled trial
Source: PLoS Med. 2019 Sep 9;16(9):e1002900. doi: 10.1371/journal.pmed.1002900 (PMC6733443; doi:10.1371/journal.pmed.1002900)
Supplement: S1 Text — QI, quality improvement. (PDF) [file pmed.1002900.s002.pdf]

S1 National consultation for developing quality improvement guideline

TECHNICAL REPORT ON MODEL FOR QUALITY IMPROVEMENT FOR STANDARD NEONATAL CARE IN HOSPITALS OF NEPAL

BASED ON TWO-DAYS' WIDER GROUP CONSULTATION WORKSHOP

Government of Nepal
Ministry of Health
Department of Health Services
Child Health Division
Kathmandu

Venue: Park Village Resort, Budhanilkantha

Date: 15-16 March 2017

ACRONYMS

CHD	Child Health Division
FHD	Family Health Division
GIZ	*Deutsche Gesellschaft für Internationale Zusammenarbeit*
H4L	Health for Life
HBB	Helping Babies Breathe
HDC	Hospital Development Committee
HMIS	Health Management Information System
ICU	Intensive Care Unit
IMNCI	Integrated Management of Neonatal and Childhood Illness
LMD	Logistics Management Division
MCH	Maternal and Child Health
MD	Management Division
MDT	Multidisciplinary Team
MPDSR	Maternal and Perinatal Death Surveillance and Review
MS	Medical Superintendent
PDSA	Plan, Do, Act and Study
PHAMED	Public Health, Monitoring and Evaluation Division
PSG	Perinatal Stakeholders Group
ROSA	Regional Office for South Asia
SDGs	Sustainable Development Goals
QI/C	Quality Improvement/Cycle
SNCU	Sick Newborn Care Unit
UNICEF	United Nations Children’s Fund
USAID	United States Agency for International Development
WHO	World Health Organization

TABLE OF CONTENTS

1.0 PROCEEDINGS OF DAY I (March 15, 2017)........1
1.1 INTRODUCTION........1
1.2 PRESENTATIONS........1
1.3 GROUP DISCUSSION/FEEDBACKS........3
1.4 DISCUSSION/FEEDBACK ON DRAFT OF IMPLEMENTATION GUIDELINE........4
1.5 REMARKS BY DR BIKASH LAMICHHANE, DIRECTOR, CHD........8
1.6 GROUP WORK AND DISCUSSION........8
2.0 PROCEEDINGS OF DAY II (March 16, 2017)........10
2.1 RISKS AND SOLUTIONS........11
2.2 FINAL CONSENSUS MODEL OF QUALITY IMPROVEMENT FOR STANDARD NEONATAL CARE IN HOSPITALS OF NEPAL........12
3.0 ANNEXES........13

1.0 PROCEEDINGS OF DAY I (March 15, 2017)

1.1 INTRODUCTION

The emcee for the workshop was Mr. ***Deepak Jha*** (IMNCI Section, Child Health Division) who started the workshop with chairing of the programme (Dr Bikash Lamichhane, Director, Child Health Division) and chief guest Dr Naresh Pratap KC (Director, Family Health Division) together with delegates from different government and non-government organisations.

1.2 PRESENTATIONS

Dr Amrit Pokhrel (IMNCI Section, Child Health Division) started the presentation session with objectives for the workshop. His presentation focused on background of neonatal health and the need for quality improvement (QI) to improve neonatal mortality and ultimately meet Sustainable Development Goals (SDGs). He further stressed that while QI has been initiated by the government, the policy remained poor and steering committees at various levels were largely non-functional.

It was followed by presentation from ***Dr Alyssa Sharkey*** (UNICEF ROSA) who presented on international guidelines and World Health Organization (WHO) *Quality of Care Framework*. Her presentation focused on standards of care and initiatives taken in different countries bases on those standards. She also provided insights on what is being done in Bangladesh for QI on maternal, neonatal and child health services.

To support the evidence, ***Mr Abhishek Gurung*** (Lifeline Nepal) did a short presentation on regional evidence of use of QI for improving maternal and neonatal care. His presentations highlighted some of the QI approaches taken within and outside Nepal to improve neonatal mortality.

Dr Ashish KC (UNICEF) presented on the fundamentals of improving quality of care using QI processes where he highlighted on the *Plan, Do, Act and Study* (PDSA) model. Moreover, he also detailed on the fundamental steps of QI processes to be implemented for improving quality of newborn care and they would unfold in the successful implementation of the interventions. He further mentioned that providing equipment doesn't guarantee service availability and service availability doesn't ensure quality of care.

Mr Dipak Raj Chaulagain (Lifeline Nepal) did a presentation on the set of activities to be conducted under the guideline. Three main stages of implementation namely, **Inception Phase, Implementation Phase** and **Sustainability Phase** are mentioned in the guidelines with a set of activities listed under them.

1.3 GROUP DISCUSSION/FEEDBACKS

Dr Naresh Pratap KC (Director, FHD) suggested to align the activities of QI done by Management Division (MD) to align with Maternal and Perinatal Death Surveillance and Review (MPDSR) committee which is currently functioning.

Dr Binamra (GIZ) gave an example of Japan where quality is a culture and they strive for delivering quality services. Helping Babies Breathe (HBB) did not work in Tanzania because it is a critical skill and people forget if they do not practice it. The scenario is similar here in Nepal too. GIZ started practice sessions in some birthing centres and orient staffs on HBB. They found that in most of the places, they did not review. The outcomes were good in only those who had kept on practising the skills. He raised the issue of accountability because the training sessions were more like coaching sessions and people did not follow the protocols after the sessions were over.

Dr Karuna Laxmi Shakya (UNICEF) shared her experiences while working in the Family Health Department (FHD). She reiterated Dr Naresh's suggestions regarding a setup of working committee for quality control in MD and below structures and to avoid duplication of committees through building a common umbrella to incorporate all activities under one belt. She also highlighted on similar interventions in hospitals in *Taplejung* and *Makwanpur* where the focus was more on maternal and child health (MCH). They established a separate hospital improvement committee to focus on MCH issues consisting of medical superintendent (MS), matron, concerned nurses, store, emergency, lab and support staffs. They conducted quarterly review meetings on maternal and neonatal health where they did self-assessments and gap identification. FHD is planning to scale up this process in other districts. Hence, it is necessary to coordinate with other divisions to avoid duplication in the districts. The intervention might be different for tertiary level hospitals due to different scope of work. ***Dr Bhadra (***H4L) also suggested for varying approaches for different level hospitals. He also reiterated the need for social audit and effective data management.

Dr Sheela Verma's comments were regarding lack of quality services despite having all the infrastructures in place. She shared her experience of a similar kind of study conducted in partnership with UNICEF among 12 hospitals. She mentioned that while *Mahendranagar* had all the equipment and logistics in place, they lacked a paediatrician. But the issue was with the

recording of data. They reviewed the charts which had no mention of time of arrival of sick children or other vital information. There is lack of motivation among staffs. The QI tools should be developed taking into consideration all the areas rather than just maternal and newborns. ***Mr Bhuwan Baral*** (Care Nepal/SUAAHARA) mentioned that QI is focused more on supply side rather than demand side and suggested that awareness among communities was necessary for better QI implementation.

1.4 DISCUSSION/FEEDBACK ON DRAFT OF IMPLEMENTATION GUIDELINE

Mr Dipak shared a draft of the QI guideline and stressed while neonatal health has been the focus of the government for a long time, two things have always been on debate –

- Quality improvement means assuring everything is in place and functioning which will ensure quality of care is maintained. There is nothing new to it and no new intervention is required.
- If quality is required, quality improvement must be focused and should be forwarded as an intervention.

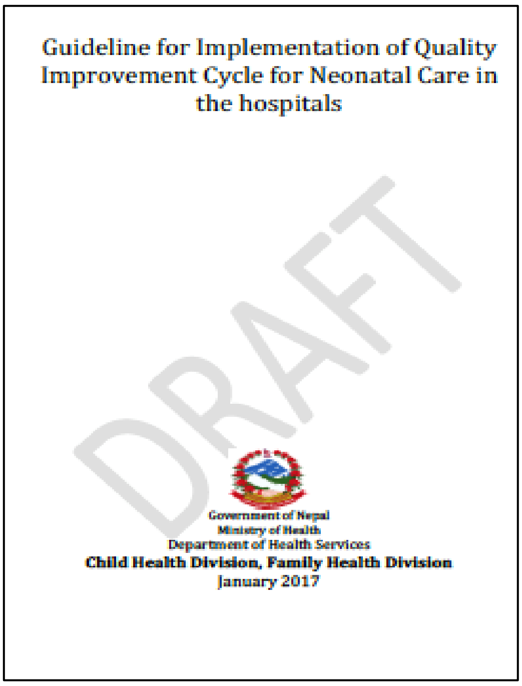

Another debate is regarding the shape of newborn services as to whether it should stay as a part of the broader maternal and neonatal health or that it should be focused as a separate entity and acted accordingly. Having said that, it cannot be conducted as a vertical programme even though extra focus is required to improve the overall status of newborns. CHD has been initiating the effort with a small number of hospitals and while all things cannot be done in a single event, the guideline will act as a reference to achieve quality in the future.

There are three phases as described before:

I. *Inception Phase (3 months)*

Mentors and internal QI facilitators will be selected in coordination with PSG and MDT at each hospital. At the end of inception phase, the hospital will prepare an on-site plan based on PDSA approach. Then, implementation phase will start together with review, gap findings, refresher trainings for any updates, management of equipment (self or through higher support) and resource mobilisation.

II. *Implementation Phase (9 months)*

This will include capacity building of health workers on QIC for neonatal care. The QIC process will be implemented together with provision of QI tools. The MDT will review the QI process on a regular basis. Refresher trainings will also be provided to health workers on regular intervals.

III. *Sustainability Phase (3 months)*

Continuous assessment of activities will be conducted together with review meetings, lesson learning sessions and way forward. The objective is that the hospitals will be able to identify their strengths and weaknesses and be able to sustain accordingly for quality improvement.

Mr Parashu Ram Shrestha (IMNCI Section Chief, CHD***)*** had some reservations regarding coverage of services. He stressed that while the focus would be among few hospitals, it should not lead to a bigger mass being left out. Hence, it should be carried out in conjunction with other divisions and relevant partners to avoid any duplication and communication gap. He further clarified regarding selection of hospitals for the intervention depending on the number of deliveries on a yearly basis. Besides, district hospitals were also selected for problem identification and issues solving at the district level in the future.

Dr Bhadra also stressed on community level intervention package. He suggested that district hospitals were different in terms of intervention and they should be managed accordingly.

Three mentors will be hired who will have extensive experience of working in child health and who will be able to facilitate the QI process from zonal to district hospitals. There will not be a parallel structure and the Perinatal Stakeholders Group (PSG) will be established as a part of

the QI committee within the hospital. The need is for a group to be established in every hospital to guide to whole QI process. ***Dr Sheela*** and ***Dr Dhana Raj Aryal*** shared a common notion regarding duplication of activities and mentioned there already was a working committee called MPDSR above district hospitals. The guidelines have already been developed and we could combine our efforts with that committee for better implementation of our activities.

All participants had a consensus on strengthening existing committee rather than creating a new one. They agreed that a QI committee was necessary to monitor and govern the activities but also insisted on using the existing committees and strengthening them rather than creating a new one. Regarding multidisciplinary team (MDT), they had different views with some agreeing on creating one while others disagreed. ***Dr Karuna*** was sceptical if it was the same for district hospitals given the low number of staffs.

Mr Dipak described about the training package for mentors and internal QI facilitators, a seven-day package which was yet to be finalised. After the training, the facilitators will go back to their working stations and start service readiness assessment, with the support from mentors. It will be followed up by equipment availability assessment, procurement and resource mobilisation. This will be done using PDSA model for on-site planning. This whole process will be completed in the inception phase. ***Dr Binamra*** had further queries regarding role of internal QI facilitators as to how they will function in the whole process to which ***Mr Dipak*** clarified that it was role of the facilitator to do the overall assessment and facilitate the QI process.

The PDSA model is based on the WHO framework and the whole approach will be based on this. The refresher training will also be based on the same model.

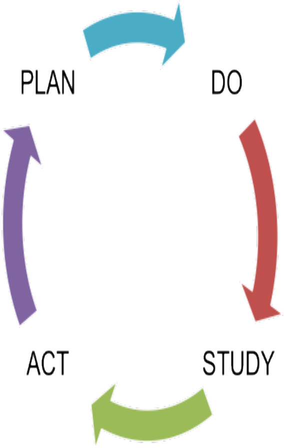

PLAN, make a clear plan for actions targeting a certain problem including assigning who is responsible, a deadline for undertaking the planned action and an expected outcome of the action.

DO, to carry out the planned action;

STUDY, to compare the expected outcome of the undertaken action with the result of the action, and;

ACT, to reflect and discuss the lessons learnt relating to the action and its outcome including taking decisions on refinements, adjustments or how to support the action to become routine.

All health workers (emergency, delivery room and SNCU/ICU) in the implementing districts will be trained on QIC approach and skills by CHD. ***Dr Sheela*** further suggested that the chair of the HDC be oriented on the guidelines for better understanding and ownership. ***Dr Binamra*** stated that health workers should be asking for training based on PDSA cycle rather than spoon feeding approach. ***Dr Ashish*** clarified that PDSA model is basically about ensuring QI process while refresher trainings are all about ensuring standards. ***Dr Bhadra*** supported ***Dr Ashish***'s comment mentioning there might be gaps which they identify while working and refresher trainings will be important platforms to address those issues.

Dr Karuna had a query regarding measurement of indicators and how the recording and reporting system, including feedback was going to work. The intervention is a new one and hence the recording and reporting will not be incorporated in the HMIS/DHIS system. ***Mr Dipak*** clarified that there would be register developed which will be used. Besides, the medical recorder/medical record officer will be used for delivery of information from the hospitals to the central level. ***Dr Bhadra*** suggested for a coordination between MD and CHD for better data management.

1.5 REMARKS BY DR BIKASH LAMICHHANE, DIRECTOR, CHD

He insisted that services should now be quality centred rather than quantity and stated that government itself is scaling up activities for newborns through setup of new sick newborn care unit (SNCU)/intensive care unit (ICU) and provision of medicines for under-five children. He urged partners for development of standards for better management and implementation of activities. He also stressed the need for behavioural change and possible ways to bring about change among health workers either through supervision/monitoring or through training. Furthermore, he suggested that blaming others for issues should stop and everyone should motivate for betterment. Finally, he stressed the need to focus on district and below structures if neonatal deaths are to be reduced and prevent overcrowding in big hospitals.

1.6 GROUP WORK AND DISCUSSION

The participants were then divided into two groups for discussion and feedback on the draft of implementation guideline and any other issues that might be seen.

Group I (Inception/Implementation Phase)	***Group II (Implementation/Sustainability Phase)***
Facilitator - Dipak Raj Chaulagain	*Facilitator - Dr Ashish KC*
Dr Bikash Lamichhane	Parashu Ram Shrestha
Dr Hemanta Ojha	Dr Sheela Verma
Dr Amrit Pokhrel	Dr Karuna Laxmi Shakya
Dr Rameshwar Man Shrestha	Dr Dhana Raj Aryal
Dr Rajendra Bhadra	Dr Kalpana
Dr Meera Upadhyaya	Dipak Jha
Chahana Singh Rana	Bhuwan Baral
Udev Maharjan	Dr Binamra Rajbhandari
Nisha Rana	
Abhishek Gurung	

***Dr Hemanta* (MD)** had issues with the formation of a new group which would run vertically and insisted on strengthening the existing structures. He reiterated that the MPDSR was functioning in the same way. Besides, he also mentioned that while the QI team might be able to function in the regional and zonal hospitals, district hospitals would not function in the same

way due to small number of staffs. ***Dr Ashish*** clarified mentioning communication channels which would be horizontal and vertical both as the internal QI facilitator would act as a messenger. However, ***Dr Hemanta*** still was not confident it was going to work without incentives. ***Dr Ashish*** further clarified incentivising did not always work and it was more about social recognition and responsibility. It was more about motivation and positive thinking. He also mentioned that it was an evidence-based approach and it had been thoroughly reviewed and implemented in many countries. Despite that, the sustainability would still be a challenge.

Dr Ashish presented hospital based study showing misclassification and low reporting of data. He also pointed the flaw in our review system where we do death review rather than process review (*unpublished data*). He insisted that the whole review should be based on QI process. The monitoring will be based on process indicators to ensure the clinical performance should be good. That should ultimately lead to survival status. This will be channelled through checklists provided to the hospitals where the staffs will fill in the forms to ensure the process is done and the outcome of the child will be written. The whole information will be displayed on the dashboard daily to ensure services are delivered. The facilitator will then check whether the process is fulfilled and quality is assured.

He then described about the eight dimensions mentioned in the WHO Standards Guideline for further clarification. Having said that, ***Dr Hemanta*** insisted on bringing aboard Health Management Information System (HMIS) citing reasons of data not being reported from bigger hospitals. ***Dr Ashish*** clarified by saying while the register is developed and will be used for recording, there is no assurance if it is going to work due to which it will take some time before it can be incorporated in the system.

2.0 PROCEEDINGS OF DAY II (March 16, 2017)

The second day continued with group discussions from earlier day. The consensus was that where there is a QI committee, the existing committee (MPDSR) will be used. ***Dr Bikash*** said the while the committee would give it a formal shape, the facilitator would be the one who would be working for the whole implementation process. He also said that the committee should be a legitimate one. It is necessary for authenticity. However, ***Dr Sheelu Adhikari*** (USAID) suggested the committee be formed at the earnest rather than waiting for governing bodies to form due to delay in implementation. She further said that while the MD did have a guideline, it has not been accelerated yet so this intervention can go ahead with the formation through the medical superintendent. ***Dr Bikash*** also agreed to her views. Besides, there is no formal guideline at the zonal and regional level so this can be a breakthrough into formalising one. Endorsement would however be necessary so coordination and meetings with MD and Curative Division would be important.

There was further discussion regarding availability of MPDSR committees in zonal and regional hospitals and consensus was developed to use the same committees to act as perinatal stakeholders' group (PSG). There will be two teams –

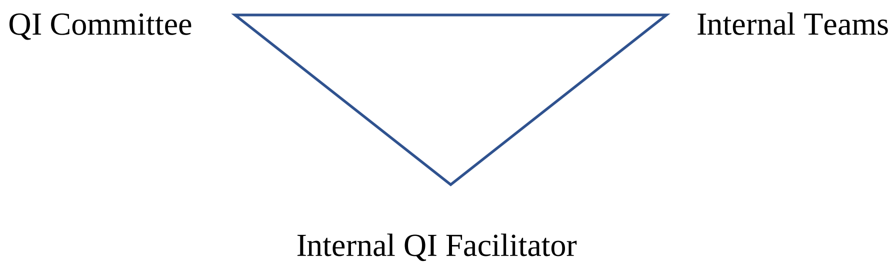

There is need for a vertical and horizontal communication to show that quality is an issue. The issues will be raised by the facilitator and managed by the QI Committee. ***Dr Bikash*** raised the issue of human resources being critical to implementation of QI process together with sustainability. Citing such reasons, he suggested the intervention to be flexible and not time bound. ***Dr Ashish*** also described about checklists which will be developed to help facilitators and staffs with timely identification of issues and deal accordingly. The registers will be kept in separate wards and they will be helpful in monitoring progress over time. There are evidences of it being helpful in developing skills and self-assessment through skills checks. CHD will provide a two-day update on clinical standards to health workers.

Dr Sheelu had further issues regarding use of new QI tools and suggested to use the tools developed by MD to which ***Dr Ashish*** cleared mentioning the same will be used and developed. He further stated that the whole idea was to focus on QI process rather than clinical outcomes. The whole thing will be reviewed through process audit rather than outcome audit. ***Dr Sheelu*** also suggested that the review/evaluation process should be simultaneously rather than on a linear basis to which everyone agreed. One of the potential risks is the staff turnover which also needs to be addressed. ***Dr Bikash*** also suggested for another meeting of relevant stakeholders (MD, CHD, FHD, LMD, Curative Division, PHAMED and other partners) for update and way forward.

2.1 RISKS AND SOLUTIONS

One of prime issues raised was who would be the secretary of the committee for this implementation process. In previous committees, the medical recorder/medical record officer is usually the secretary. The group had some discussions regarding scope of work and decided that the committee guidelines and protocols should be followed to avoid any clashes in the future. Some of the participants had different opinions regarding medical recorders as they may not adhere to guidelines citing extra work. ***Dr Sheelu*** shared her previous experiences where the medical recorders did not adhere in MPDSR meetings. The consensus was that while the records would be kept by the medical recorders, facilitators will be the one guiding the process. The intervention is not trying to build a new structure rather strengthen the existing one.

Risks	Possible causes	Solutions
Feedback loop not functional at the central level	Lack of dedicated human resources to compile, review and provide feedback on the data	Feedback unit at CHD
Interoperability of IT infrastructures at the district and central levels		
Inadequate response by the central ministry on improving the quality of care		
Financial resources for MPDSR meetings		

The two-day workshop was formally closed by ***Dr Amrit Pokhrel*** on behalf of CHD.

2.2 FINAL CONSENSUS MODEL OF QUALITY IMPROVEMENT FOR STANDARD NEONATAL CARE IN HOSPITALS OF NEPAL

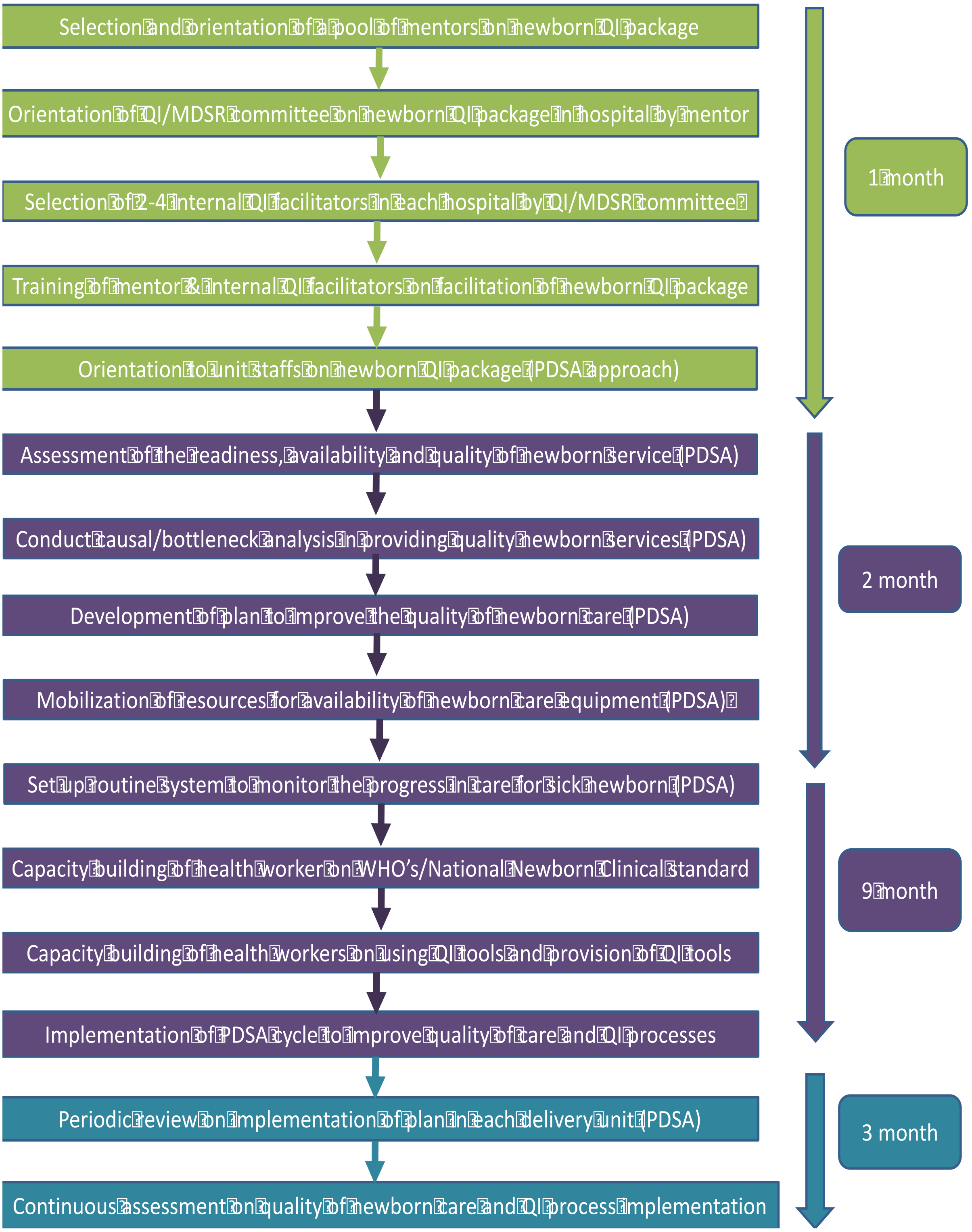

3.0 ANNEXES

Quality Improvement Cycle for Neonatal Care

(IMPLEMENTATION GUIDELINE FINALIZATION)

Next Steps with timeline and responsibility

Point of Contact: ***Dipak Raj Chaulagain***

Activities	Responsible person	Timeline
Consolidated workshop report	Abhishek Gurung	20 March
Register (Recording and Reporting)<br>Sick newborn (Guideline)	Dipak Chaudhary<br>Dipak Raj Chaulagain	24 March
QI Tools (Six + KMC) - Guideline	Dipak Raj Chaulagain	24 March
Assessment tools (readiness form)	Dipak Chaudhary	24 March
TOR of QI committee, MPDR committee, mentors, internal facilitators, internal unit teams	Dipak Raj Chaulagain	21 March
Roles and responsibilities – hospital, RHD, CHD, FHD, CD, PHAMED, MD, *Monitoring Cell*	Dipak Raj Chaulagain	22 March
PDSA action plan tools (On-site planning guideline)	Udev Maharjan	22 March
Clinical protocol	Dr Amrit Pokhrel	22 March
Operational guideline	Dr Amrit Pokhrel	22 March
LOG Frame, data collection indicators and tools	Dr Ashish KC<br>Abhishek Gurung	20 March
Background, schematic diagram	Dipak raj Chaulagain	19 March
Consolidated meeting with third version of QI guideline	Dipak Raj Chaulagain	26 March
Joint meeting with CHD, FHD, MD, CD, PHAMED	Dipak Chaulagain<br>Dr. Ashish KC	28 March
Finalized implementation guideline		31 March
Endorsement of the implementation guideline for ***Quality Improvement Cycle for neonatal care***		

Quality Improvement Cycle for Neonatal Care in hospital of Nepal
Implementation Guideline Finalization Workshop
15-16 March 2017

Objectives of the workshop

1. To build consensus on implementation approach of Quality Improvement Cycle for neonatal care in hospitals.
2. To finalize interventions of Quality Improvement Cycle for neonatal care in hospitals.
3. To finalize the Implementation Guideline: Quality Improvement Cycle for neonatal care

Day 1-15 March, 2017		
8.00-8.45	***Breakfast***	
8.45-9.15	Registration, Opening, Introduction	
9.15-9.30	Objective of the workshop	Parashu Ram Shrestha, IMNCI Section Chief, CHD
9.30-10.00	Need of QI in hospital settings to improve quality of care in Nepal	Dr. Amrit Pokhrel
10.00-10.30	Global experience on QI implementation for improving neonatal care	Dr. Alyssa Sharkey, UNICEF ROSA
10.30-11.00	Fundamentals of improving Quality of Care through QI processes<br>Regional evidence on QI for newborn care	Dr. Ashish, UNICEF<br><br>Abhishek Gurung, Lifeline Nepal
11.00-11.30	Remarks	Dr. Bikash Lamichhane, Director, CHD<br>Dr. Naresh Pratap KC, Director, FHD
11.30-12.00	Tea Break	
12.00- 1.15	Sharing of draft QIC guideline (implementation approach, implementation phases, Interventions)	Dipak Chaulagain, Lifeline Nepal
1.15-2.15	***Lunch***	
2.15-2.30	Group Division and assignment of task	Dipak Chaulagain<br>Abhishek Gurung
2.30-5.30	Group Work	Group
Day 2-16 March, 2017		
8.00-8.30	***Breakfast***	
8.30-9.30	*Group work continue (including preparation for presentation)*	***Group***
9.30-11.30	Group Work sharing	Groups
11.30-12.00	Tea Break	
12.00-1.30	Discussion on major changes in draft QIC implementation guideline	Dr. Amrit/Dipak Chaulagain
1.30-2.30	Lunch	
2.30-3.30	First wave/phase of implementing the guideline	Dr. Amrit Pokhrel, CHD
3.30-4.30	Planning and way forward	Parashu Ram Shrestha, IMNCI Section Chief, CHD
4.30-4.45	Closing	Dr. Bikash Lamichhane, Director Child Health Division

PARTICIPANTS AT THE CONSULTATION WORKSHOP, PARK VILLAGE, BUDHANILAKANTHA (MARCH 15-16, 2017)
