# Supplementary material for: Effect of a scaled-up neonatal resuscitation quality improvement package on intrapartum-related mortality in Nepal: A stepped-wedge cluster randomized controlled trial
Source: PLoS Med. 2019 Sep 9;16(9):e1002900. doi: 10.1371/journal.pmed.1002900 (PMC6733443; doi:10.1371/journal.pmed.1002900)
Supplement: S3 Text — SOP, standard operating protocol. (PDF) [file pmed.1002900.s004.pdf]

S4 Data collection and management SOP

NePeriQIP

Standard Operating Protocol for
Data Collection, Management and Quality Assurance

Aim

The aim of the standard operating protocol (SOP) is to provide guidance to data collectors, clinical observers, research coordinators and research manager on the data collection, management and data quality assurance.

Study period

NePeriQIP will collect data at the study sites between 1 July 2017 and 30 September 2019. Retrospective data collection for the months of April 14 and June 2017 will be done based on medical registries.

Data collection method

The data collection for NePeriQIP project will be done using different methods to assess different outcome measures as mentioned in the study protocol.

These methods include

1. Data extraction - Retrieval of data from individual patient journals as well as registers in labour room and sick newborn ward.
2. Exit interview - Interview with the mothers at the time of pre-discharge
3. Clinical observation - Clinical observation of the delivery care in the labour room

Table 1. Data collection structure

	Forms	Who	When	Where
Data extraction	Mother and newborn data retrieval form (Form 1a and 1b)	Data collector	Daily basis	Labour room
	Sick newborn data retrieval form (Form 4)	Data collector	Daily basis	Sick newborn ward
	Neonatal Encephalopathy form (Form 5)	Data collector	Daily basis	Sick newborn ward
Exit interview	Client exit interview form (Form 2a and 2b)	Data collector	Daily basis before discharge	At postnatal ward
	Social autopsy form (Form 3a and 3b)	Data collector	Daily basis before discharge	At postnatal ward
Clinical observation	Clinical observation form (Form 6)	Clinical observer	Daily basis in every birth	At labour room

Table 2. Places where the type of data collection

S.N	Hospital	Form 1	Form 2	Form 3	Form 4	Form 5	Form 6
1	Western Regional Hospital (Pokhara)	✓	✓	✓	✓	✓	✓
2	Mid-Western Regional Hospital (Surkhet)	✓	✓	✓	✓		
3	Bardiya district hospital	✓	✓	✓	✓		
4	Bharatpur Hospital	✓	✓	✓	✓	✓	✓
5	Seti Zonal Hospital	✓	✓	✓	✓		
6	Nuwakot district hospital	✓	✓	✓	✓		
7	Koshi Zonal Hospital	✓	✓	✓	✓	✓	✓
8	Rapti Sub Regional Hospital (Dang)	✓	✓	✓	✓		
9	Nawalparasi district hospital	✓	✓	✓	✓		
10	Lumbini Zonal Hospital	✓	✓	✓	✓	✓	✓
11	Bheri Zonal Hospital	✓	✓	✓	✓		
12	Pyuthan district hospital	✓	✓	✓	✓		

A. <u>DATA COLLECTION PROCESS</u>

1. NePeriQIP Surveillance registry (see Annex 1)

1.1 On a daily basis, in the morning, the data collector needs to go to the admission room in delivery unit and SNCU/NICU to get the information of newly admitted women and neonates. The following information from the admission record should be transferred to the surveillance registry: inpatient ID provided by the hospital, name of the mother, date & time of admission (column 1,3 and 4). A study ID should also be assigned according to protocol (column 2) (see below).

1.2 On a daily basis, in the afternoon at 2:00 pm, the data collector need to go to the medical record section and fill in the surveillance registry on the outcome of patient visit (column 5-9) the date and time of discharge (column 10).

1.3 On a daily basis, collect all completed forms and tick columns 11-22 as appropriate. File all completed forms.

1.4 Check all forms not completed and follow-up as possible.

1.5 On a weekly basis on Sunday 4:00 pm, the data collector needs to fill in the weekly summary report and submit it together with all completed forms to the database manager. Also take a copy of the surveillance registry for the forms submitted and attach to the weekly summary report.

2. Mother and newborn data extraction form (Form 1)

2.1 On a daily basis, after filling in the surveillance registry, the data collector will fill in one copy of Form 1a (1b for multiple deliveries) for the deliveries that were conducted in the delivery unit since last data collection until 8:00 am of the current day. Data should be retrieved from the client journals.

2.2 Use the study ID provided in the surveillance registry.

2.3 If there is a multiple birth (twin or triplet), use Form 1b.

2.4 If the client journals are not available, assess the place of referral of the mother either PNC or other ward, then go to the place and fill in the Form 1.

2.5 After the patient is discharged, complete the Form 1 and provide the signature on the front page of the form.

2.6 Put the Form 1 in the appropriate folder once you have initiated to fill up Form 1.

2.7 On a weekly basis, please provide the information on the completed Form 1 in the surveillance registry.

3. Sick newborn data retrieval form (Form 4)

3.1 After the completion of the Form 1, the data collector needs to fill up Form 4.

3.2 Form 4 needs to be filled up for all the babies who are sick and admitted in the hospital on 28 day or less.

3.3 Use the study ID provided in the surveillance registry.

3.4 Complete the Form 4 in the medical record room after discharge of the sick baby and sign the completed Form 4.

3.5 Put Form 4 the appropriate folder once you have initiated to fill up Form 4.

3.6 On a weekly basis, please provide the information on the completed Form 4 in the surveillance registry.

4. Neonatal Encephalopathy Form (Form 5)

4.1 This is for the four hospitals where there are clinical observers (see Table 2).

4.2 After the completion of Form 4, the data collector needs to fill up Form 5 if Apgar score at 5 minutes is below 6.

4.3 Examination and Thompson scoring will be conducted by medical officer (MO), who will note the results in the medical record.

4.4 Form 5 needs to be filled up only for babies referred from delivery unit, i.e. babies born at the hospital.

4.5 Please use the same study ID used in Form 1, if the baby was delivered in the same hospital.

4.6 Put the Form 5 in the appropriate folder once you have initiated to fill up Form 5.

4.7 Complete Form 5 in the medical record room after discharge of the sick baby and sign the completed Form 5.

4.8 On a weekly basis, please provide information on all completed Form 5 in the surveillance registry.

5. **Client Exit interview (Form 2)**

5.1 On a daily basis, in the morning after filling in the surveillance registry, communicate with staff to list all mothers due for discharge.

5.2 Check that Form 1 has been completed for all mothers due for discharge.

5.3 Identify and locate mothers due for discharge.

5.4 Once the mothers have been identified and located, please provide information about the study and take consent from the client to conduct exit interview.

5.5 After providing the information and taking consent from the mother, conduct an exit interview with the mother using Form 2a (if multiple delivery use Form 2b).

5.6 Interviews should be conducted before mothers leave the hospital. If a mother has left or absconded, mark the form as missing in surveillance registry.

5.7 Put Form 2 in the appropriate folder once you have completed to fill it up.

5.8 On a weekly basis, please provide information on all completed Form 2 in the surveillance registry.

6. **Social autopsy Form (Form 3)**

6.1 On a daily basis after the filling up the surveillance registry on the mothers who had delivered a baby between last data collection to current day 8 am, assess whether there have been any stillbirths or neonatal deaths since last data collection.

6.2 If there was a stillbirth or a neonatal death, identify where the mother has been transferred to, PNC or other room.

6.3 Similarly, based on the review of the sick newborn register in the hospital, if mothers with neonatal death are identified, find where they are located.

6.4 Once the mother has been identified and located, please provide brief counselling to the mother on the loss of the baby.

6.5 After the counselling, provide information about the study.

6.6 If the mother agrees to participate in the study, take a written consent from the mother.

6.7 After providing the information and taking consent from the mother, conduct the social autopsy interview with the mother, or a relative if the mother is unavailable, using Form 3a (if multiple delivery use Form 3b).

6.8 Interview should be conducted before mother/relative leaves the hospital. If mother/relative has left or absconded, mark the form as missing in surveillance registry.

6.9 Put Form 3 in the appropriate folder once you have completed to fill it up.

6.10 On a weekly basis, please provide information on all completed Form 3 in the surveillance registry.

7. **Clinical observation Form (Form 6)**

7.1 This is for the four hospitals where there are clinical observers in the labour room.

7.2 Once the women enter the delivery room, register woman's name and inpatient ID (601-603. Leave the study ID blank.

7.3 Once the mothers have been identified and Form 6 has been initiated, please provide information about the study and take consent from the client or relative for doing observation during delivery.

7.4 Observe all the steps being provided in the labour room in Form 6.

7.5 After the observation has been completed, please provide your signature to the form 6.

7.6 When 1 hour has passed after delivery, or after the patient has been transferred to PNC, complete Form 6 and provide your signature on the form.

7.7 Give all Form 6 to the data collector in charge of coordinating data collection. This data collector will then fill in the accurate study ID based on surveillance registry on the form in order to complete it.

7.8 Put the completed Form 6 in the appropriate folder.

7.9 On a weekly basis, please provide the information on all completed Form 6 in the surveillance registry.

B. <u>DATA MANAGEMENT PROCESS</u>

1. After completing each form, the data collector needs to review the completeness of the form

For Form 1, 4 and 5

2. If Form 1 and 4 (information collected via data extraction) are being filled, assess the completeness of form immediately after filling it up.
3. Review the information which has not been filled, again do a review of the client journal and add the information if available.
4. If the required information is not available in the client journal, then assess the registry and fill up the information.
5. Check that Mother's name (101 and 102) and inpatient ID (103) are the same in Forms 4 (401- 403) with the corresponding study ID.

For Form 2 and 3

6. If Form 2 and 3 (information collected via interview) are being filled, assess the completeness of the form immediately after filling it up.
7. Please ensure that the contact information of the care-giver as well as her closest family information.

8. Review the information which has not been filled, again ask the questions which has not been answered.
9. If the required information is not available in Form 2 and 3 make a call to the mother to assess the information.
10. Check that Mother's name and Medical Record ID in Form 1 (101-103) are the same in Forms 2 (201- 203) and 3 (301-303) with the corresponding study ID.

For Form 6

11. If Form 6 (information collected via observation) is being filled, assess the completeness of the form immediately after the delivery.
12. Check that Mother's name and inpatient ID in Form 1 (101-103) are the same in Form 6 (601- 603) with the corresponding study ID.

C. DATA QUALITY ASSURANCE PROTOCOL

1. On the weekly basis at the time of compilation, following important information needs to be assessed and reported from the surveillance registry in the weekly summary report (Annex 2):
   a. Total number of admission of women for delivery
   b. Total number of women delivering in the hospital
   c. Total Form 1 filled
   d. Total Form 2 filled
   e. Total Form 3 filled (For stillbirth and neonatal death)
   f. Total number of sick newborn admitted in sick newborn ward
   g. Total Form 4 filled (for sick newborn)
   h. Total form 5 filled (for sick newborn)
   i. Total Form 6 filled (only for 4 hospitals)

2. The above information needs to be assessed from surveillance registry

D. ASSIGNMENT OF STUDY ID

Unique study IDs are essential to a successful data collection. The study ID is different from medial record ID or any other ID that patients might be assigned when coming to hospital. The NePeriQIP study IDs are unique for this study.

Study IDs will contain six digits. The first two digits are identifiers for each hospital. Hospitals with large number of deliveries will have two identifying numbers. The remaining four digits is an order number, starting at 0001.

- ***Study IDs should be assigned in order.***
- ***Only one mother-baby pair can have a certain study ID.***
- ***Study IDs can never be reassigned.***

In case there is a multiple delivery, ***no new study ID is assigned*** by the data collector. Instead Forms 1b and 2b should be used and the extra information generated due to multiple delivery will be handled at data entry by data manger at research office.

When a baby that has not been delivered at the hospital is admitted to the SNCU/NICU a new study ID will be assigned to the mother/guardian of the baby. If twins not born at the hospital are admitted they will get the same study ID. ***The study ID is always connected to the mother/guardian!***

Study IDs are divided as follows on the different hospital:

	**Hospitals**	**ID numbers**
1	Western Regional Hospital	100001-129999
2	Mid-Western Regional Hospital	130000-149999
3	Bardiya district hospital	150000-159999
4	Bharatpur Hospital	200000-229999
5	Seti Zonal Hospital	230000-249999
6	Nuwakot district hospital	250000-259999
7	Koshi Zonal Hospital	300000-329999
8	Rapti Sub Regional Hospital	330000-349999
9	Nawalparasi district hospital	350000-359999
10	Lumbini Zonal Hospital	400000-429999
11	Bheri Zonal Hospital	430000-449999
12	Pyuthan district hospital	450000-459999

Annex 1: NePeriQIP Surveillance Registry

Name of hospital: ____________________________________ **Registry order number: __________________**

1	**Inpatient Number**								
2	**Study ID**								
3	**Name of the mother**								
4	**Date of Admission (dd/mm/yy)**								
5	**Outcome of admission**								
6	**Date of Delivery (dd/mm/yy) or N/A**								
7	**Outcome of the baby**								
8	**In-born sick newborn baby (tick)**								
9	**Out-born sick newborn baby (tick)**								
10	**Date of discharge (dd/mm/yy)**								
11	**Form 1 initiated (tick)**								
12	**Form 1 completed (tick)**								
13	**Form 2 initiated (tick)**								
14	**Form 2 completed (tick)**								
15	**Form 2 no interview (tick)**								
16	**Form 3 initiated (tick)**								
17	**Form 3 completed (tick)**								
18	**Form 3 no interview (tick)**								
19	**Form 4 initiated (tick)**								
20	**Form 4 completed (tick)**								
21	**Form 5 initiated (tick)**								
22	**Form 5 initiated (tick)**								
23	**Form 6 initiated (tick)**								
24	**Form 6 completed (tick)**								
25	**Sent to office (tick)**								

Outcome of admission: 1= Vaginal Delivery, 2= Caesarean section, 3= Admitted to SNCU, 9=Went home

Outcome of the baby: 1=alive at discharge, 2=stillbirth and 3=neonatal death

Annex 2.

NePeriQIP Weekly Summary Report

Name of the hospital: ______________________________

Name and ID of data collector submitting report: ______________________________

Reporting period (date – date): ______________________________

Date report sent from hospital (BS) (dd/mm/yyyy): ______________________________

Total number of admission of women for delivery	
Total number of women delivering in the hospital	
Total Form 1a and 1b not yet completed (*not included in submission*)	
Total Form 1a and 1b completed and submitted	
Total Form 2a and 2b not yet completed (*not included in submission*)	
Total Form 2a and 2b completed and submitted	
Total Form 2a and 2b without interview	
Total Form 3a and 3b not yet completed (*not included in submission*)	
Total Form 3a and 3b completed and submitted	
Total Form 3a and 3b without interview	
Total Form 4 not yet completed (*not included in submission*)	
Total Form 4 completed and submitted	
Total number of sick newborn admitted in SNCU/NICU	
Total Form 5* not yet completed (*not included in submission*)	
Total Form 5* completed and submitted	
Total form 6* not yet completed	
Total Form 6* completed	

*only for Western Regional Hospital, Bharatpur Hospital, Koshi Zonal Hospital and Lumbini Zonal Hospital
