# Supplementary material for: Effect of a scaled-up neonatal resuscitation quality improvement package on intrapartum-related mortality in Nepal: A stepped-wedge cluster randomized controlled trial
Source: PLoS Med. 2019 Sep 9;16(9):e1002900. doi: 10.1371/journal.pmed.1002900 (PMC6733443; doi:10.1371/journal.pmed.1002900)
Supplement: S1 Table — (PDF) [file pmed.1002900.s005.pdf]

S1 Table. Timeline of initiation of intervention in each hospital

	Intended intervention start	Actual intervention start
**Wedge 1**		
Western Regional Hospital	17-Aug-2017	19-Aug-2017
Midwestern Regional Hospital	17-Aug-2017	6-Aug-2017
Bardiya District Hospital	17-Aug-2017	1-Sept-2017
**Wedge 2**		
Bharatpur Zonal Hospital	17-Nov-2017	16-Nov-2017
Seti Zonal Hospital	17-Nov-2017	10-Nov-2017
Nuwakot District Hospital	17-Nov-2017	20-dec-2017
**Wedge 3**		
Koshi Zonal Hospital	13-Feb-2018	6-Feb-2018
Rapti Sub-regional Hospital	13-Feb-2018	24-May-2018
Nawalparasi District Hospital	13-Feb-2018	23-Feb-2018
**Wedge 4**		
Lumbini Zonal Hospital	15-May-2018	9-Jul-2018
Bheri Zonal Hospital	15-May-2018	5-Jul-2018
Pyuthan District Hospital	15-May-2018	24-May-2018
**TOTAL**		

Table 2. Background characteristics in the control and intervention period (as per protocol)

	Control (N-40139, % with CI)	Intervention (N-48875, % with CI)	Total (N-89014, % with CI)	p-value
Maternal age (Mean±SD)	23.9±4.3	24.1±4.4	24.0±4.3	
Disadvantaged Ethnic group	56.5% (56.0-57.0)	56.5% (56.1-56.9)	56.5% (56.2-56.8)	0.967
Illiterate	4.1% (3.9-4.3)	3.9% (3.6-4.0)	4.0% (3.9-4.1)	0.02
Parity				
Nullipara	45.5 (45.0-46.0)	45.3 (44.9-45.7)	45.4 (45.0-45.8)	0.586
Primipara	34.9 (34.4-35.4)	35.3 (34.9-35.7)	35.1 (34.7-35.5)	0.143
Multipara	19.6 (19.2-20.0)	19.3 (19.0-19.6)	19.4 (19.1-19.7)	0.244
Sex of baby				
Boy	53.6 (53.1-54.1)	54.3 (53.9-54.7)	45.4 (45.0-45.8)	0.03
Girl	46.4 (45.9-46.9)	45.7 (46.3-47.1)	35.1 (34.7-35.5)	0.03
Mode of delivery*				
Normal vaginal	76.0 (75.6-76.4)	73.1 (72.7-73.5)	74.4 (74.1-74.7)	<0.001
Assisted vaginal	3.9 (3.7-4.1)	3.9 (3.7-4.1)	3.9 (3.8-4.0)	0.846
Emergency C-section	13.9 (13.6-14.2)	15.5 (15.2-15.8)	14.8 (14.6-15.0)	<0.001
Elective c-section	5.4 (5.2-5.6)	6.5 (6.3-6.7)	6.0 (5.8-6.2)	<0.001
Not recorded	0.8 (0.7-0.9)	0.8 (0.7-0.9)	0.8 (0.7-0.9)	
Complication during labour	3.3 (3.1-3.5)	3.2 (3.0-3.4)	3.2 (3.1-3.3)	0.577
Gestational age (mean ± SD)	38.1±2.6	38.2±2.6	38.2±2.6	
Gestational age categorized				
37 weeks and more	83.2 (82.8-83.6)	83.1 (82.8-83.4)	83.2 (83.0-83.4)	0.632
Less than 37 weeks	16.8 (16.4-17.2)	16.9 (16.6-17.2)	16.8 (16.6-17.0)	0.632
Birth weight (mean±SD)	2795.9±474.2	2778.2±466.9	2786.3±470.3	
Birth weight categorized**				
Normal birth weight (≥ 2500 gram)	82.8 (82.4-83.2)	83.0 (82.7-83.3)	82.9 (82.7-83.1)	0.46
Low birth weight (<2500 gram)	17.2 (16.8-17.6)	17.0 (16.7-17.3)	17.1 (16.9-17.3)	0.46

*missing-820, **missing-50

Table 3a. Intrapartum related mortality rate in control and intervention period (as per protocol) using GLMM

	**Control**		Intervention		aOR	CI 95%	p-value	ICC
	Deaths/Deliveries	Rate per 1000 deliveries, (95% CI)	Deaths/Deliveries	Rate per 1000 deliveries, (95% CI)				
Intrapartum related mortality	430/40139	10.7 (9.7-11.8)	376/48875	7.7 (6.9-8.5)	0.79	0.69-0.92	0.002	0.0286
Intrapartum stillbirth	281/40139	7.0 (6.2-7.9)	218/48875	4.5 (3.9-5.1)	0.73	0.61-0.88	<0.001	0.1160
First day mortality	149/39858	3.7 (3.2-4.4)	158/48657	3.2 (2.8-3.8)	0.92	0.73.-1.16	0.49	0.1557
Early neonatal mortality	502/39858	12.6 (11.5-13.7)	488/48657	10.0 (9.2-11.0)	0.89	0.78-1.02	0.09	0.1538

Table 4a. Intrapartum related mortality rates (deaths/deliveries) in control and intervention periods (ITT) by wedge, hospital size and individual hospitals. (Per protocol)

	Deaths/births	Rate per 1000 births, (95% CI)	Deaths/births	Rate per 1000 births, (95% CI)	p-value	cOR (95% CI)	aOR (95% CI)[1]
Wedge 1	54/4341	12.5 (9.4-16.1)	94/13372	7.0 (5.6-8.6)	0.001	0.56 (0.40-0.79)	0.69 (0.49-0.97)[2]
Wedge 2	93/11791	7.7 (6.2-9.5)	97/19268	5.1 (4.2-6.2)	0.004	0.64 (0.48-0.85)	0.67 (0.51-0.90)
Wedge 3	99/9132	10.7 (8.6-13.2)	69/7644	9.4 (7.5-11.6)	0.384	0.83 (0.61-1.13)	0.82 (0.60-1.12)
Wedge 4	184/14875	12.5 (10.8-14.5)	116/8591	13.2 (101.0-15.7)	0.673	1.09 (0.87-1.38)	0.99 (0.78-1.26)[2]
Volume hospital	Control	Rate	Intervention	Rate	p-value	cOR (95% CI)	aOR (95% CI)
High volume	259/22772	11.3 (9.9-12.7)	215/28944	7.6 (6.6-8.6)	<0.001	0.65 (0.54-0.78)	0.66 (0.55-0.80)
medium volume	119/13785	8.4 (6.9-10.1)	141/16885	8.5 (7.2-10.0)	0.91	0.97 (0.76-1.24)	0.97 (0.76-1.24)
low volume	52/3582	15.2 (11.3-19.9)	20/3046	6.7 (4.2-9.9)	0.001	0.45 (0.27-0.75)	0.45 (0.27-0.77)
By hospital	Control	Rate	Intervention	Rate	p-value	cOR (95% CI)	aOR (95% CI)
Hospital 1	35/3079	11.4 (8.0-15.8)	52/7649	6.8 (5.1-8.9)	0.016	0.60 (0.39-0.92)	0.74 (0.48-1.15)
Hospital 2	16/1002	15.1 (8.8-224.0)	40/4841	8.3 (5.9-11.3)	0.035	0.51 (0.29-0.92)	0.66 (0.36-1.20)
Hospital 3	3/260	13.9 (2.9-40.1)	2/882	2.2 (0.3-7.8)	0.019	0.20 (0.03-1.18)	0.27 (0.04-1.75)
Hospital 4	59/6780	8.6 (6.5-11.1)	68/11239	6.1 (4.7-7.7)	0.05	0.69 (0.49-0.98)	0.71 (0.50-1.00)
Hospital 5	25/4090	5.9 (3.8-8.6)	24/7025	3.5 (2.2-5.2)	0.07	0.56 (0.31-0.98)	0.63 (0.36-1.12)
Hospital 6	9/921	10.5 (5.6-20.8)	5/1004	5.1 (1.9-11.1)	0.168	0.51 (0.17-1.52)	0.60 (0.19-1.86)
Hospital 7	49/4719	10.3 (7.6-13.6)	39/5006	7.9 (5.6-10.7)	0.20	0.75 (0.49-1.14)	0.74 (0.48-1.12)
Hospital 8	41/3753	10.6 (7.4-15.4)	30/2258	12.7 (9.4-17.1)	0.46	1.22 (0.76-1.96)	1.15 (0.71-1.87)
Hospital 9	9/660	14.1 (4.1-26.9)	0/400	0.0 (0.0-0.0)	<0.001	-	-
Hospital 10	116/8194	14.2 (11.7-17.1)	56/5050	11.3 (8.7-14.4)	0.137	0.78 (0.57-1.08)	0.69 (0.50-0.96)
Hospital 11	37/4940	7.9 (5.5-10.8)	47/2761	15.7 (11.5-20.8)	0.001	2.30 (1.49-3.54)	2.19 (1.42-3.39)
Hospital 12	31/1741	17.8 (12.0-25.3)	13/780	16.8 (9.2-28.0)	0.857	0.94 (0.49-1.80)	0.79 (0.40-1.55)

[1] forward modelling test for mode of delivery, preterm birth and sex of baby

[2] adjusted for preterm birth
