# Supplementary material for: Effect of a scaled-up neonatal resuscitation quality improvement package on intrapartum-related mortality in Nepal: A stepped-wedge cluster randomized controlled trial
Source: PLoS Med. 2019 Sep 9;16(9):e1002900. doi: 10.1371/journal.pmed.1002900 (PMC6733443; doi:10.1371/journal.pmed.1002900)
Supplement: S2 Table — GLMM, generalized linear mixed model. (PDF) [file pmed.1002900.s006.pdf]

S2 Table. Intrapartum related mortality GLMM analysis as per protocol

	**Control**		Intervention		aOR	CI 95%	p-value	ICC
	Deaths/Deliveries	Rate per 1000 deliveries, (95% CI)	Deaths/Deliveries	Rate per 1000 deliveries, (95% CI)				
Intrapartum related mortality	430/40139	10.7 (9.7-11.8)	376/48875	7.7 (6.9-8.5)	0.79	0.69-0.92	0.002	0.0286
Intrapartum stillbirth	281/40139	7.0 (6.2-7.9)	218/48875	4.5 (3.9-5.1)	0.73	0.61-0.88	<0.001	0.1160
First day mortality	149/39858	3.7 (3.2-4.4)	158/48657	3.2 (2.8-3.8)	0.92	0.73.-1.16	0.49	0.1557
Early neonatal mortality	502/39858	12.6 (11.5-13.7)	488/48657	10.0 (9.2-11.0)	0.89	0.78-1.02	0.09	0.1538
