# Supplementary material for: Effect of a scaled-up neonatal resuscitation quality improvement package on intrapartum-related mortality in Nepal: A stepped-wedge cluster randomized controlled trial
Source: PLoS Med. 2019 Sep 9;16(9):e1002900. doi: 10.1371/journal.pmed.1002900 (PMC6733443; doi:10.1371/journal.pmed.1002900)
Supplement: S3 Table — (PDF) [file pmed.1002900.s007.pdf]

S3 Table. Intrapartum mortality sub-group analysis per protocol

	Deaths/births	Rate per 1000 births, (95% CI)	Deaths/births	Rate per 1000 births, (95% CI)	p-value	cOR (95% CI)	aOR (95% CI)[1]
Wedge 1	54/4341	12.5 (9.4-16.1)	94/13372	7.0 (5.6-8.6)	0.001	0.56 (0.40-0.79)	0.69 (0.49-0.97)[2]
Wedge 2	93/11791	7.7 (6.2-9.5)	97/19268	5.1 (4.2-6.2)	0.004	0.64 (0.48-0.85)	0.67 (0.51-0.90)
Wedge 3	99/9132	10.7 (8.6-13.2)	69/7644	9.4 (7.5-11.6)	0.384	0.83 (0.61-1.13)	0.82 (0.60-1.12)
Wedge 4	184/14875	12.5 (10.8-14.5)	116/8591	13.2 (101.0-15.7)	0.673	1.09 (0.87-1.38)	0.99 (0.78-1.26)[2]
Volume hospital	Control	Rate	Intervention	Rate	p-value	cOR (95% CI)	aOR (95% CI)
High volume	259/22772	11.3 (9.9-12.7)	215/28944	7.6 (6.6-8.6)	<0.001	0.65 (0.54-0.78)	0.66 (0.55-0.80)
medium volume	119/13785	8.4 (6.9-10.1)	141/16885	8.5 (7.2-10.0)	0.91	0.97 (0.76-1.24)	0.97 (0.76-1.24)
low volume	52/3582	15.2 (11.3-19.9)	20/3046	6.7 (4.2-9.9)	0.001	0.45 (0.27-0.75)	0.45 (0.27-0.77)
By hospital	Control	Rate	Intervention	Rate	p-value	cOR (95% CI)	aOR (95% CI)
Hospital 1	35/3079	11.4 (8.0-15.8)	52/7649	6.8 (5.1-8.9)	0.016	0.60 (0.39-0.92)	0.74 (0.48-1.15)
Hospital 2	16/1002	15.1 (8.8-224.0)	40/4841	8.3 (5.9-11.3)	0.035	0.51 (0.29-0.92)	0.66 (0.36-1.20)
Hospital 3	3/260	13.9 (2.9-40.1)	2/882	2.2 (0.3-7.8)	0.019	0.20 (0.03-1.18)	0.27 (0.04-1.75)
Hospital 4	59/6780	8.6 (6.5-11.1)	68/11239	6.1 (4.7-7.7)	0.05	0.69 (0.49-0.98)	0.71 (0.50-1.00)
Hospital 5	25/4090	5.9 (3.8-8.6)	24/7025	3.5 (2.2-5.2)	0.07	0.56 (0.31-0.98)	0.63 (0.36-1.12)
Hospital 6	9/921	10.5 (5.6-20.8)	5/1004	5.1 (1.9-11.1)	0.168	0.51 (0.17-1.52)	0.60 (0.19-1.86)
Hospital 7	49/4719	10.3 (7.6-13.6)	39/5006	7.9 (5.6-10.7)	0.20	0.75 (0.49-1.14)	0.74 (0.48-1.12)
Hospital 8	41/3753	10.6 (7.4-15.4)	30/2258	12.7 (9.4-17.1)	0.46	1.22 (0.76-1.96)	1.15 (0.71-1.87)
Hospital 9	9/660	14.1 (4.1-26.9)	0/400	0.0 (0.0-0.0)	<0.001	-	-
Hospital 10	116/8194	14.2 (11.7-17.1)	56/5050	11.3 (8.7-14.4)	0.137	0.78 (0.57-1.08)	0.69 (0.50-0.96)
Hospital 11	37/4940	7.9 (5.5-10.8)	47/2761	15.7 (11.5-20.8)	0.001	2.30 (1.49-3.54)	2.19 (1.42-3.39)
Hospital 12	31/1741	17.8 (12.0-25.3)	13/780	16.8 (9.2-28.0)	0.857	0.94 (0.49-1.80)	0.79 (0.40-1.55)

[1] forward modelling test for mode of delivery, preterm birth and sex of baby

[2] adjusted for preterm birth
